# Subthalamic nucleus shows opposite functional connectivity pattern in Huntington’s and Parkinson’s disease

**DOI:** 10.1093/braincomms/fcad282

**Published:** 2023-12-06

**Authors:** Stefania Evangelisti, Sirius Boessenkool, Chris Patrick Pflanz, Romina Basting, Jill F Betts, Mark Jenkinson, Stuart Clare, Kinan Muhammed, Campbell LeHeron, Richard Armstrong, Johannes C Klein, Masud Husain, Andrea H Nemeth, Michele T Hu, Gwenaëlle Douaud

**Affiliations:** FMRIB Centre, Wellcome Centre for Integrative Neuroimaging, John Radcliffe Hospital, University of Oxford, OX3 9DU Oxford, UK; Nuffield Department of Clinical Neurosciences, University of Oxford, OX3 9DU Oxford, UK; Department of Biomedical and Neuromotor Sciences, University of Bologna, 40127 Bologna, Italy; FMRIB Centre, Wellcome Centre for Integrative Neuroimaging, John Radcliffe Hospital, University of Oxford, OX3 9DU Oxford, UK; Nuffield Department of Clinical Neurosciences, University of Oxford, OX3 9DU Oxford, UK; FMRIB Centre, Wellcome Centre for Integrative Neuroimaging, John Radcliffe Hospital, University of Oxford, OX3 9DU Oxford, UK; Nuffield Department of Clinical Neurosciences, University of Oxford, OX3 9DU Oxford, UK; Stroke Research Group, Department of Clinical Neuroscience, University of Cambridge, CB2 0QQ Cambridge, UK; FMRIB Centre, Wellcome Centre for Integrative Neuroimaging, John Radcliffe Hospital, University of Oxford, OX3 9DU Oxford, UK; Nuffield Department of Clinical Neurosciences, University of Oxford, OX3 9DU Oxford, UK; Department of Experimental Psychology, University of Oxford, OX2 6GG Oxford, UK; FMRIB Centre, Wellcome Centre for Integrative Neuroimaging, John Radcliffe Hospital, University of Oxford, OX3 9DU Oxford, UK; Nuffield Department of Clinical Neurosciences, University of Oxford, OX3 9DU Oxford, UK; FMRIB Centre, Wellcome Centre for Integrative Neuroimaging, John Radcliffe Hospital, University of Oxford, OX3 9DU Oxford, UK; Nuffield Department of Clinical Neurosciences, University of Oxford, OX3 9DU Oxford, UK; School of Computer Science, Faculty of Engineering, University of Adelaide, 5005 Adelaide, Australia; FMRIB Centre, Wellcome Centre for Integrative Neuroimaging, John Radcliffe Hospital, University of Oxford, OX3 9DU Oxford, UK; Nuffield Department of Clinical Neurosciences, University of Oxford, OX3 9DU Oxford, UK; Nuffield Department of Clinical Neurosciences, University of Oxford, OX3 9DU Oxford, UK; Nuffield Department of Clinical Neurosciences, University of Oxford, OX3 9DU Oxford, UK; New Zealand Brain Research Institute, 8011 Christchurch, New Zealand; Nuffield Department of Clinical Neurosciences, University of Oxford, OX3 9DU Oxford, UK; FMRIB Centre, Wellcome Centre for Integrative Neuroimaging, John Radcliffe Hospital, University of Oxford, OX3 9DU Oxford, UK; Nuffield Department of Clinical Neurosciences, University of Oxford, OX3 9DU Oxford, UK; FMRIB Centre, Wellcome Centre for Integrative Neuroimaging, John Radcliffe Hospital, University of Oxford, OX3 9DU Oxford, UK; Nuffield Department of Clinical Neurosciences, University of Oxford, OX3 9DU Oxford, UK; Department of Experimental Psychology, University of Oxford, OX2 6GG Oxford, UK; Nuffield Department of Clinical Neurosciences, University of Oxford, OX3 9DU Oxford, UK; Nuffield Department of Clinical Neurosciences, University of Oxford, OX3 9DU Oxford, UK; FMRIB Centre, Wellcome Centre for Integrative Neuroimaging, John Radcliffe Hospital, University of Oxford, OX3 9DU Oxford, UK; Nuffield Department of Clinical Neurosciences, University of Oxford, OX3 9DU Oxford, UK

**Keywords:** subthalamic nucleus, differential effect, functional connectivity, Huntington’s, Parkinson’s

## Abstract

Huntington’s and Parkinson’s disease are two movement disorders representing mainly opposite states of the basal ganglia inhibitory function. Despite being an integral part of the cortico-subcortico-cortical circuitry, the subthalamic nucleus function has been studied at the level of detail required to isolate its signal only through invasive studies in Huntington’s and Parkinson’s disease. Here, we tested whether the subthalamic nucleus exhibited opposite functional signatures in early Huntington’s and Parkinson’s disease. We included both movement disorders in the same whole-brain imaging study, and leveraged ultra-high-field 7T MRI to achieve the very fine resolution needed to investigate the smallest of the basal ganglia nuclei. Eleven of the 12 Huntington’s disease carriers were recruited at a premanifest stage, while 16 of the 18 Parkinson’s disease patients only exhibited unilateral motor symptoms (15 were at Stage I of Hoehn and Yahr off medication). Our group comparison interaction analyses, including 24 healthy controls, revealed a differential effect of Huntington’s and Parkinson’s disease on the functional connectivity at rest of the subthalamic nucleus within the sensorimotor network, i.e. an opposite effect compared with their respective age-matched healthy control groups. This differential impact in the subthalamic nucleus included an area precisely corresponding to the deep brain stimulation ‘sweet spot’—the area with maximum overall efficacy—in Parkinson’s disease. Importantly, the severity of deviation away from controls’ resting-state values in the subthalamic nucleus was associated with the severity of motor and cognitive symptoms in both diseases, despite functional connectivity going in *opposite* directions in each disorder. We also observed an altered, opposite impact of Huntington’s and Parkinson’s disease on functional connectivity within the sensorimotor cortex, once again with relevant associations with clinical symptoms. The high resolution offered by the 7T scanner has thus made it possible to explore the complex interplay between the disease effects and their contribution on the subthalamic nucleus, and sensorimotor cortex. Taken altogether, these findings reveal for the first time non-invasively in humans a differential, clinically meaningful impact of the pathophysiological process of these two movement disorders on the overall sensorimotor functional connection of the subthalamic nucleus and sensorimotor cortex.

## Teaser

Using MRI at 7T, Evangelisti *et al*. report that participants with early Huntington’s and Parkinson’s disease show opposite pattern of resting-state functional connectivity in the subthalamic nucleus and sensorimotor cortex. Despite going in opposite direction, functional connectivity values in each disorder showed pathological associations with motor and cognitive symptoms.

## Graphical Abstract

**Graphical Abstract:**
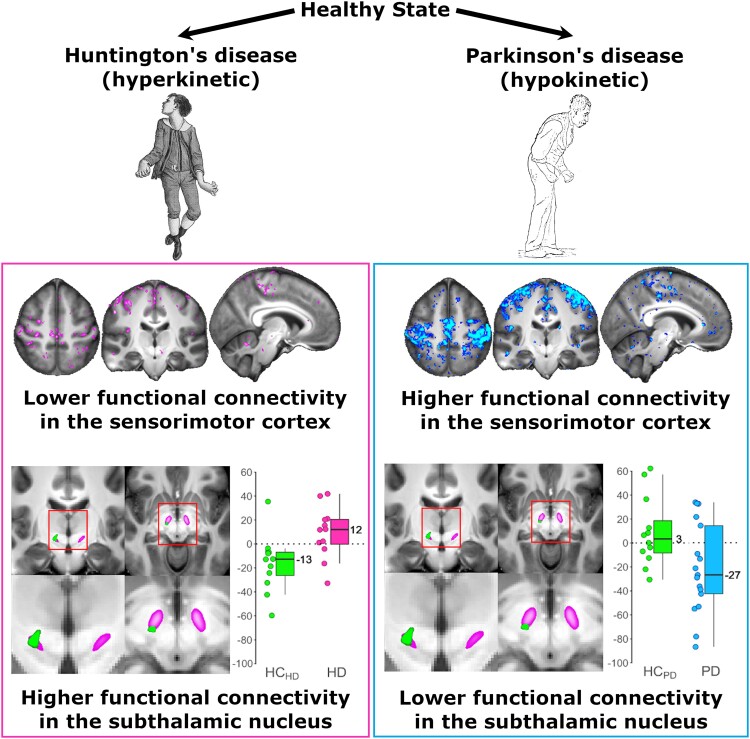

## Introduction

The basal ganglia receive massive convergence of inputs from cortical areas involved in movement, learning and reward systems. In particular, motor connections follow two competing pathways linking cortex and thalamus, the indirect and the direct pathways.^1,2^ The smallest of all the basal ganglia structures, the subthalamic nucleus (STN), plays a crucial role in motor function not only as a key element of the indirect pathway but also of the additional so-called hyperdirect loop, which conducts information faster than both direct and indirect pathways.^3,4^ Both indirect and hyperdirect pathways suppress movement by elevating the inhibitory basal ganglia output, while the direct pathway promotes it.^5^ A functional imbalance between direct and indirect pathways, but also possibly the hyperdirect one, is thought to explain part of the opposing motor phenotypes observed in two key basal ganglia disorders—Huntington’s disease (HD) and Parkinson’s disease (PD)—with the former primarily associated with hyperkinetic movements, such as chorea, and the latter characterized by hypokinetic signs.

HD is a fatal, autosomal dominant neurodegenerative disease that initially predominantly affects the GABAergic medium-size spiny neurons of the striatum, leading to choreic movements.^6^ PD, on the other hand, is a progressive neurological disorder characterised by a degeneration of dopaminergic neurons in the substantia nigra (SN) pars compacta that manifests itself with various motor symptoms, one of the most characteristic of which is bradykinesia, a decremental slowness of movement.^7^

To date, only invasive studies have been able to functionally investigate the STN in HD and PD at the level of detail required to identify this subcortical structure and, crucially, to distinguish it from the adjacent SN. The exact functional role of the STN in PD, explored in numerous studies of deep brain stimulation (DBS) in patients with advanced disease, remains unclear, however. Inhibition, decorrelation and even elevation of its activity all improve motor symptoms, suggesting it is an abnormal pattern of activity that lies at the heart of this movement disorder.^8^ Conversely, while degeneration of the STN in HD has been reported more than four decades ago,^9^ evidence for its functional involvement in HD is scarcer in humans. Studies on different HD animal models have, however, recently demonstrated an early impairment of spontaneous STN activity.^10-13^

Conventional non-invasive measures in human, such as MRI at 1.5 or 3T, usually lack the resolution and contrast to achieve such a key distinction, typically with voxels inferior to 1.5 mm isotropic in size. Improvements gained by using state-of-the-art 7 T provide a unique opportunity to isolate the signal from the STN^14-16^ and to study its spontaneous neuronal fluctuations (‘functional connectivity’) using resting-state functional MRI (rs-fMRI).^17^

Here, we investigated the functional connectivity of the STN and sensorimotor cortex in *both* HD and PD at high resolution using 7T MRI. Including the two movement disorders in the same study makes it possible for the first time to *directly* compare in humans the three states of basal ganglia inhibition: decreased (HD), increased (PD) and normal (healthy controls, HC) at an unprecedented level of detail. The main aims of our study were 2-fold: first, to assess whether we could detect in the STN, and the sensorimotor cortex, the theoretical opposite states in basal ganglia dysfunction for HD and PD; second, whether these differential functional signatures could be related to clinical symptoms in HD and PD.

## Materials and methods

### Participants

The study was approved by the local Research Ethics Committee (South Central—Oxford A) and written consent was obtained from each participant (recruited 2014–20).

We aimed to include participants early in the course of the disease to limit confounding effects, and to keep the groups as homogenous as possible to maximize the detection of differences. We primarily included in this study participants with premanifest HD on the one hand, and unilateral PD participants at Hoehn and Yahr—H&Y—Stage I off medication on the other hand.^18^

Exclusion criteria pertained to the safety risks specific to the 7T scanner, such as dental implants, head/neck/shoulder tattoos, or any surgery where vascular clips could have been used (without post-operative imaging to formally eliminate the possibility of their presence).

#### Huntington’s disease (HD) carriers

Twelve HD carriers were recruited through the HD clinic at the Oxford University Hospitals (OUH). To characterize motor and cognitive symptoms, a trained neurologist administered the Unified Huntington’s Disease Rating Scale (UHDRS)^19^ and the Hopkins verbal learning test (HLVT).^20^ Following amended criteria described in the TRACK-HD study,^21,22^ the HD carriers were classified based on a combination of total motor score (TMS), total functional capacity (TFC) and diagnostic confidence score, which are subscales of the UHDRS. Eleven participants were at a premanifest stage, and one participant showed manifest HD; Table 1 shows demographics and summary clinical information for the HD participants (more details in Supplementary Table 1).

**Table 1:** Summary demographic and clinical measures for Huntington’s disease (HD) carriers, Parkinson’s disease (PD) patients and healthy controls, before (HC) and after splitting them into groups matched to HD and PD (HC_HD_ and HC_PD_)

HD carriers			PD patients		
*N*		12	*N*		18
Premanifest		11 (92%)	Disease duration (months from diagnosis)		33.1 ± 27.4 (1–89)
Manifest		1 (8%)	H&Y (Off)		1.17 ± 0.38 (1–2)
CAG expansion repeats ^a^		42.2 ± 2.7 (37–46)		Stage 1	15 (83%)
Disease-burden score ^a^		250.7 ± 119.0 (87–413)		Stage 2	3 (17%)
UHDRS:	Total Motor Score	10.3 ± 10.5 (1–27)	UPDRS-III (Off)		20.3 ± 8.0 (9 −39)
	Total Functional Capacity	11.9 ± 2.2 (6–13)	Medicated for PD ^b^	Yes	15 (83%)
	Diagnostic confidence	1.5 ± 1.2 (0–4)		No	3 (17%)
	Total behavioural score ^c^	3.0 ± 4.0 (0–14)	Symptom laterality	Unilateral	16 (89%)
	Total cognitive score ^c^	321.7 ± 95.6 (199–439)		Bilateral	2 (11%)
**Demographics**					
Group	HC	HC_HD_	HC_PD_	HD	PD
*N*	25	12	13	12	18
Gender (M/F)	16 (64%) / 9 (36%)	7 (58%) / 5 (42%)	9 (69%) / 4 (31%)	5 (42%) / 7 (58%)	12 (67%) / 6 (33%)
Age (years)	45.7 ± 16.8 (21–77)	35.6 ± 14.7 (21–69)	55.3 ± 13.4 (29–77)	39.8 ± 11.4 (26–58)	59.2 ± 8.1 (47–72)
Handedness	23 R / 2 L	10 R / 2 L	13 R	8 R / 4 L	14 R / 4 AMB

UHDRS, unified Huntington’s disease rating scale; UPDRS, unified Parkinson’s disease rating scale; H&Y, Hoehn and Yahr; AMB, ambidextrous.

^a^CAG and disease burden available for all HD carriers but one.

^b^9/15 PD patients medicated with levodopa.

^c^Total behavioural/cognitive score are, respectively, the sum of all the behavioural/cognitive item scores.

#### Parkinson’s disease patients

Eighteen early-stage PD participants were recruited through the Oxford Parkinson’s Disease Centre (OPDC) and the Parkinson’s clinic at the OUH. A trained neurologist administered the Movement Disorders Society-Unified Parkinson’s Disease Rating Scale (MDS-UPDRS Parts I–IV) to quantify motor and non-motor symptoms.^23^ Table 1 shows demographics and summary clinical information for the PD participants (additional details in Supplementary Table 1). Fifteen participants were classified as H&Y Stage 1, and three as Stage 2, when off medication. Fifteen were medicated, of whom nine with levodopa. All PD patients were scanned in the morning, between 9 and 10 am. If medicated, they were scanned withdrawn from medication, having taken their last dose the night before, ∼12 h prior to clinical assessment and MRI scanning. Prior to scanning they were clinically assessed (off medication if medicated), and all of them were classified as being tremor-dominant, except one participant who was indeterminate, and one who was classified as having postural instability and gait disturbance. Laterality of motor symptoms was determined with an asymmetry index calculated from UPDRS-III motor items.^24^ While 2 participants showed bilateral motor symptoms, the vast majority—16 of the PD patients—were unilateral (9 on the left, 7 on the right).

Comparing the 3 unmedicated PD participants with the 15 medicated ones, other than differences inherent to their medication status, there was no significant difference between the two groups (under heteroscedastic assumptions) except, at an uncorrected level, in: (i) daytime sleepiness (*P* = 0.0013 uncorrected), constipation (*P* = 0.048 uncorrected) and light headedness on standing (*P* = 0.041 uncorrected), as well as neck rigidity (*P* = 0.0013 uncorrected) and right hand movements (*P* = 0.00074 uncorrected) (all unmedicated PD patients experienced no issue) and (ii) postural tremor of the left hand (all 3 unmedicated PD patients had tremor versus 7 out of the 15 medicated PD patients, *P* = 0.0013 uncorrected).

#### Healthy controls

Twenty-five HC participants were recruited for comparison with the HD and PD groups. Demographics for the HC group are reported in Table 1.

### Neuroimaging

#### Acquisition

All MR imaging took place at the Functional Magnetic Resonance Imaging of the Brain (FMRIB) centre, part of the Wellcome Centre for Integrative Neuroimaging, using a 7T Magnetom syngo B17 scanner with a 32-channel head coil (Siemens Medical Solutions, Erlangen, Germany).

The MRI protocol included, amongst other sequences:

- Two whole-brain high-resolution volumetric structural scans:
  (i) a T1-weighted image, acquired using a magnetization-prepared rapid gradient echo (MPRAGE) sequence: sagittal orientation, matrix 256 × 256, field of view (FOV) of 240 mm, 176 slices per slab, 0.94 × 0.94 × 0.94 mm^3^ resolution, inversion time (TI) of 1050 ms, echo time (TE) of 2.84 ms and repetition time (TR) of 2200 ms, flip angle of 7°, bandwidth of 240 Hz/Px, generalized autocalibrating partial parallel acquisition (GRAPPA) acceleration with factor 2;
  (ii) a PD-weighted image, also acquired with an MPRAGE sequence: sagittal orientation, matrix 256 × 256, FOV of 240 mm, 176 slices per slab, 0.94 × 0.94 × 0.94 mm^3^ resolution, TE of 2.84 ms and TR of 1240 ms, flip angle of 7°, bandwidth of 240 Hz/Px, GRAPPA acceleration with factor 2.
- Whole-brain diffusion-weighted high-resolution imaging, which was performed using an echo planar imaging (EPI) sequence: axial orientation, matrix 160 × 160, FOV of 192 mm, 104 slices, 1.2 × 1.2 × 1.2 mm^3^ resolution, TE of 68.2 ms, TR of 5382 ms, flip angle of 90°, bandwidth of 1488 Hz/Px, GRAPPA acceleration with factor 2, multi-band acceleration factor 2, 64 isotropically distributed diffusion gradient directions, b-value of 1000 s/mm^2^. Two non-diffusion-weighted imaging scans were acquired with opposite phase encoding directions (A/P and P/A).
- A 7-minute high-resolution rs-fMRI scan acquired with a gradient EPI sequence: axial orientation, A/P phase encoding direction, matrix 160 × 160, FOV of 192 mm, 104 slices, 1.2 × 1.2 × 1.2 mm^3^ resolution, TE of 25 ms, TR of 1853 ms, flip angle of 40°, bandwidth of 1644 Hz/Px, GRAPPA acceleration with factor 2, multi-band acceleration factor 4, 220 volumes. Subjects were instructed to keep their eyes open but no fixation cross was shown.

#### Image preprocessing

Image preprocessing was performed using tools from the FMRIB software library (FSL^25-27^; https://fsl.fmrib.ox.ac.uk/fsl/fslwiki).

##### Structural pre-processing

To improve contrast-to-noise ratio and image inhomogeneities, T1 images were divided by PD images within subject. For this, we registered PD to the corresponding T1 scan with a rigid registration (6 degrees of freedom). The resultant T1/PD image was used as the main structural image for processing, and as the ‘native’ space for each subject’s other imaging modalities. It was brain extracted, and then segmented into grey matter (GM),^28^ accounting for high-intensity outlier voxels (around vessels) with a combination of upper thresholding and the use of an additional tissue class.

##### Resting-state functional MRI pre-processing

First, we needed to create a map to unwarp the rs-fMRI scans. In lieu of a fieldmap, we estimated the displacement field from blip-up-blip-down pairs of diffusion images to correct for rs-fMRI distortions.^29,30^ Rs-fMRI was motion-corrected,^31^ then unwarped using the diffusion-derived fieldmap. There was no large head motion, with a maximum mean relative displacement of 0.5 mm, and no difference between the groups (on average, HC: 0.15 mm, HD: 0.15 mm, PD: 0.14 mm). Temporal filtering was performed with a high-pass filter (cut-off at 100 s). These functional images were then brain extracted. Denoising of rs-fMRI was performed with single-subject independent component analysis (ICA)^32^: we performed a manual classification of components into ‘signal’, ‘unknown’ and ‘noise’, and noise components were regressed out.^33-35^ Finally, functional images were linearly registered to the corresponding T1/PD ‘native’ space using boundary-based registration approach.^36^

##### Registration to standard space

Optimized registration to the Montreal Neurological Institute (MNI) standard space was carried out with a multi-step approach optimizing the alignment across subjects based on GM information. First, registration from T1/PD native space to MNI was performed using FSL-VBM.^37^ GM maps of a subset of participants (equal numbers from each HD, PD and HC groups) were linearly registered to a GM prior template in MNI space. By taking the mean of these images and their flipped counterparts along the *x*-axis, an initial symmetrical template in MNI space was created. The same steps were repeated with non-linear registration to the initial template to create an improved symmetrical, study-specific and unbiased template. Last, all subjects’ GM images were non-linearly registered to the study-specific template to create structural-to-MNI warpfields. Exclusion masks for the signal dropout region in structural images due to inhomogeneities in the B_1_ transmit field of the coil (mainly around a right temporo-cerebellar area) were created specifically for each participant and used in both linear and non-linear registrations to improve the alignment.

Boundary-based registration matrices from functional to native structural space, and warpfields from structural to study-specific standard space were then combined to transform the resting-state images with a single interpolation. Therefore, GM from structural T1/PD and rs-fMRI was *de facto* in the same study-specific standard space.

#### Regions-of-interest

Both for visualization, and to create probabilistic regions-of-interest (ROIs), individual, subject-specific masks were manually drawn for the STN. Thanks to the sufficient contrast and high resolution of the rs-fMRI data, STN and SN could be clearly distinguished directly on each subject’s average functional image (over the pre-processed volumes, *n* = 220, to increase signal-to-noise ratio [SNR]), after unwarping and motion-correction (Supplementary Fig. 1). Typically, these structures were first delineated in coronal view, then adjusted on axial and on sagittal views. We then checked in 3D the smoothness of the shape of the ROI. The online Human Brain atlas (http://www.thehumanbrain.info/brain/sections.php) was used as anatomical reference.

We also delineated the SN, and external and internal globus pallidus (GPe and GPi) as additional, informative ROIs of the basal ganglia circuitry that can be difficult to isolate from one another. For the manual segmentation of the GPe and GPi, we used each subject’s structural image, as the medial medullary lamina—separating GPi from GPe—was clearly visible on the T1/PD images (Supplementary Fig. 1). The GPe and GPi were first drawn on the axial view, then adjusted on sagittal and coronal views.

All the manually drawn ROIs were registered to the study-specific standard space, applying corresponding spatial transformations described above, and specific thresholding after interpolation (0.4 for STN and SN, 0.6 for GPe and GPi). The ROIs were then averaged to get probabilistic group reference masks for each region.

Finally, a large mask of the sensorimotor cortex was created by combining probabilistic masks from the Harvard Oxford atlas^38^ for the post-central and pre-central gyri (at a 5% probability threshold) and the supplementary motor area (at a 20% probability threshold).

#### Group-ICA and dual-regression analysis

Data analyses were performed using FSL, R and SPSS.

Group-ICA was run on rs-fMRI data to create 30 independent components (ICs).^32^ The same number of participants from each group was included to ensure an unbiased decomposition. Following a dual-regression approach,^39^ all 30 ICs—10 of which were identified as resting-state networks, RSNs—were first regressed into rs-fMRI data to obtain a participant-specific timecourse for each IC above and beyond all other ICs. Second, timecourses were regressed into the imaging data to obtain subject-specific spatial maps, which could then be compared between groups. This dual-regression approach inherently accounts for potential differences in baseline signal that might exist across subjects.

While the 30 group-ICs were created using the data smoothed with a Gaussian kernel of 3 mm full width at half maximum (FWHM)—below the recommended maximum 4 mm FWHM smoothing that guarantees that the majority of the signal in the centre of the STN originates from the STN itself, as opposed to from the SN^15^—output timecourses from the first regression were regressed into *unsmoothed* data to retain as much of the initial spatial resolution as possible.

**Figure 1:**
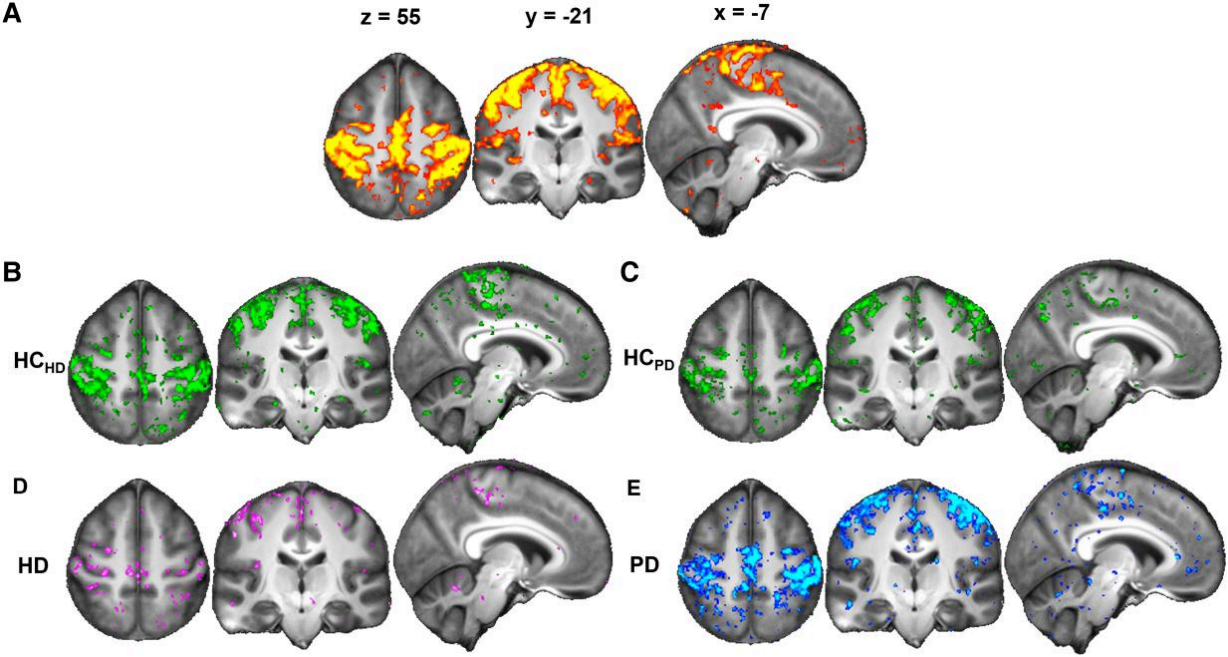
**Average functional connectivity in the sensorimotor network.** (**A**) The sensorimotor template map resulting from group-ICA is shown in red-yellow (*P* < 0.05). (**B–E**) The corresponding sensorimotor *t*-value map for each individual group average (Student’s *t* > 2.3) are shown: healthy control group matched to the HD group (HC_HD_) in green (**B**), healthy control group matched to the PD group (HC_PD_) group in green **(C)**, Huntington’s disease (HD) carriers in pink **(D)** and Parkinson’s disease (PD) participants in blue (**E**). Radiological orientation (left is right).

All subsequent analyses, i.e. group comparisons and correlations, focus on the primary sensorimotor network component that was identified amongst the 10 RSNs (Fig. 1).

### Statistical analysis

#### Group comparisons

Due to inherent baseline differences in age between our HD carriers and PD patients groups (*P* = 0.003, Tukey), HC participants were manually split into two groups so that they were age-matched to each HD and PD group: HC_HD_ and HC_PD_. There were subsequently no significant differences in age between HD and HC_HD_ (*P* = 0.62, Tukey), or PD and HC_PD_ (*P* = 0.78, Tukey). No significant differences in sex existed before (χ^2^(2) = 2.9, *P* = 0.24) or after (χ^2^(3) = 3.5, *P* = 0.32) splitting the HC group.

Our final statistical design therefore used four groups: HD participants, PD participants, HD-matched healthy controls HC_HD_, and PD-matched healthy controls HC_PD_.

As our main aim was to establish whether we could detect in the STN the opposite state in basal ganglia dysfunction for HD and PD, we investigated the following two interaction contrasts, revealing regions where HD and PD differed from their respective control groups in *opposite* ways:

1. (HD−HC_HD_)−(PD−HC_PD_)
−(HD−HC_HD_)+(PD−HC_PD_)

Permutation testing (5000 permutations) was performed on the dual-regression spatial maps using the above contrasts, as well as a mean contrast for each group individually.^40^ To reduce further the influence of age differences, age was added in the general linear model as a regressor of no interest.

In particular, STN clusters were considered significant using false discovery rate (FDR) after correction for multiple comparisons across the voxels making up the probability group mask (created by thresholding at 20%), using the uncorrected *P*-values generated by the step described above. For completeness and future replicability,^41^ we also reported results and effect sizes for uncorrected *P* < 0.001 in our additional basal ganglia ROIs.

Separately, multiple comparison correction was performed using threshold-free cluster enhancement (TFCE) as test statistic within the non-parametric permutation tool^40,42^ in the sensorimotor cortical ROI, as this method intrinsically favours larger ROIs. Results were considered significant for TFCE-corrected *P* < 0.05.

Plots were created using the weighted averages from the (1−*P*)-value maps to get parameter estimates in supra-threshold clusters of interest within our ROIs of the STN (as well as SN, GPe and GPi) and of the sensorimotor cortex. The presence of potential outliers (defined as outside ±3 interquartile range, from the median) was verified in the weighted averages of parameter estimates extracted from both full GM (automatically created for each subject by FSL-VBM) and the full group-level ICA map of our entire sensorimotor network, controlling for age in the regression. One HC and one PD were excluded from further imaging analyses following these procedures, without any issue to the matching (see Supplementary Table 2 for demographics and summary clinical information once these two participants were excluded).

#### Correlation of clinical scores with neuroimaging data

Clinical assessment scores were correlated with weighted averages extracted from supra-threshold clusters of interest from the dual-regression analysis (parameter estimates, in arbitrary units).

In particular, for HD participants, and excluding those scores that were constant for all participants or non-null only for the one manifest HD patient, these were:

- CAG number of repeats (Cytosine, Adenine, Guanine)
- Disease burden (CAG-Age-Product, or CAP, the product of excess CAG length and age^43^)
- Diagnostic confidence
- UHDRS—Motor assessment: all sub-scores, and TMS (sum of all UHDRS motor items)
- UHDRS—Functional capacity: all sub-scores
- UHDRS—Functional assessment scale
- UHDRS—Independence scale
- UHDRS—Cognitive assessment: all sub-scores
- UHDRS—Behaviour assessment: all sub-scores
- HVLT (trial 1, 2, 3, delayed recall)

Separately, for PD participants, excluding the scores that were null for all patients, these were:

- Disease duration
- UPDRS Part I: all sub-scores
- UPDRS Part II: all sub-scores
- UPDRS Part III: all sub-scores
- UPDRS Part IV: all sub-scores
- Composite scores:

1. For tremor: sum of scores for II-tremor, III-postural tremor of hands, III-kinetic tremor of hands, III-rest tremor amplitude, III-constancy of rest tremor.
2. For posture and gait: sum of scores for II-walking and balance, III-freezing, III-gait, III-freezing of gait, III-postural stability.
3. For rigidity: sum of scores for III-rigidity of neck, right and left upper and lower extremities.
4. For right and left motor symptoms, separately: sum of scores for III-rigidity of upper and lower extremities, finger tapping, hand movements, pronation/supination movements of hands, toe tapping, leg agility, postural tremor of hands, kinetic tremor of hands, rest tremor amplitude of upper and lower extremities.
5. For axial motor symptoms: sum of scores for III-speech, facial expression, rigidity neck, arising from chair, gait, freezing of gait, postural stability, posture, body bradykinesia, rest tremor amplitude of lip/jaw.

Rho correlations were evaluated with a two-sided Spearman’s rank test, and their significance was determined in *R* (using algorithm AS 89, function stats::cor.test, with exact option set to ‘true’). We applied an FDR correction, which holds under dependency, to these *P*-values. Considering the small sample sizes in both HD and PD groups, results were considered significant at a threshold of 10% FDR, corrected across all clinical and behavioural measures. For completeness, all correlations with uncorrected-*P* < 0.05 are also reported.

## Results

### Anatomical specificity of the data

Using state-of-the-art 7T, we were able to gather data at a very fine resolution crucial to study small structures that play a key role in both movement disorders and cannot be easily disentangled from one another at lower resolution (STN from SN, GPi from GPe). Supplementary Fig. 1 highlights the anatomical features that allowed us to distinguish these basal ganglia structures and draw specific ROIs for each participant.

### Rs-fMRI sensorimotor network

The main sensorimotor network that resulted from group-ICA comprised the upper limbs (arm/elbow/wrist/fist/fingers) and lower limbs (thighs/feet/toes) regions of the primary cortex, medial and posterior areas of the premotor cortex, and primary and secondary somatosensory cortex (Fig. 1A).^44^ The average functional connectivity of the sensorimotor network within each of the four groups (HC_HD_, HC_PD_, HD and PD) is shown in Fig. 1B–E.

### Rs-fMRI sensorimotor network group comparisons

**Figure 2:**
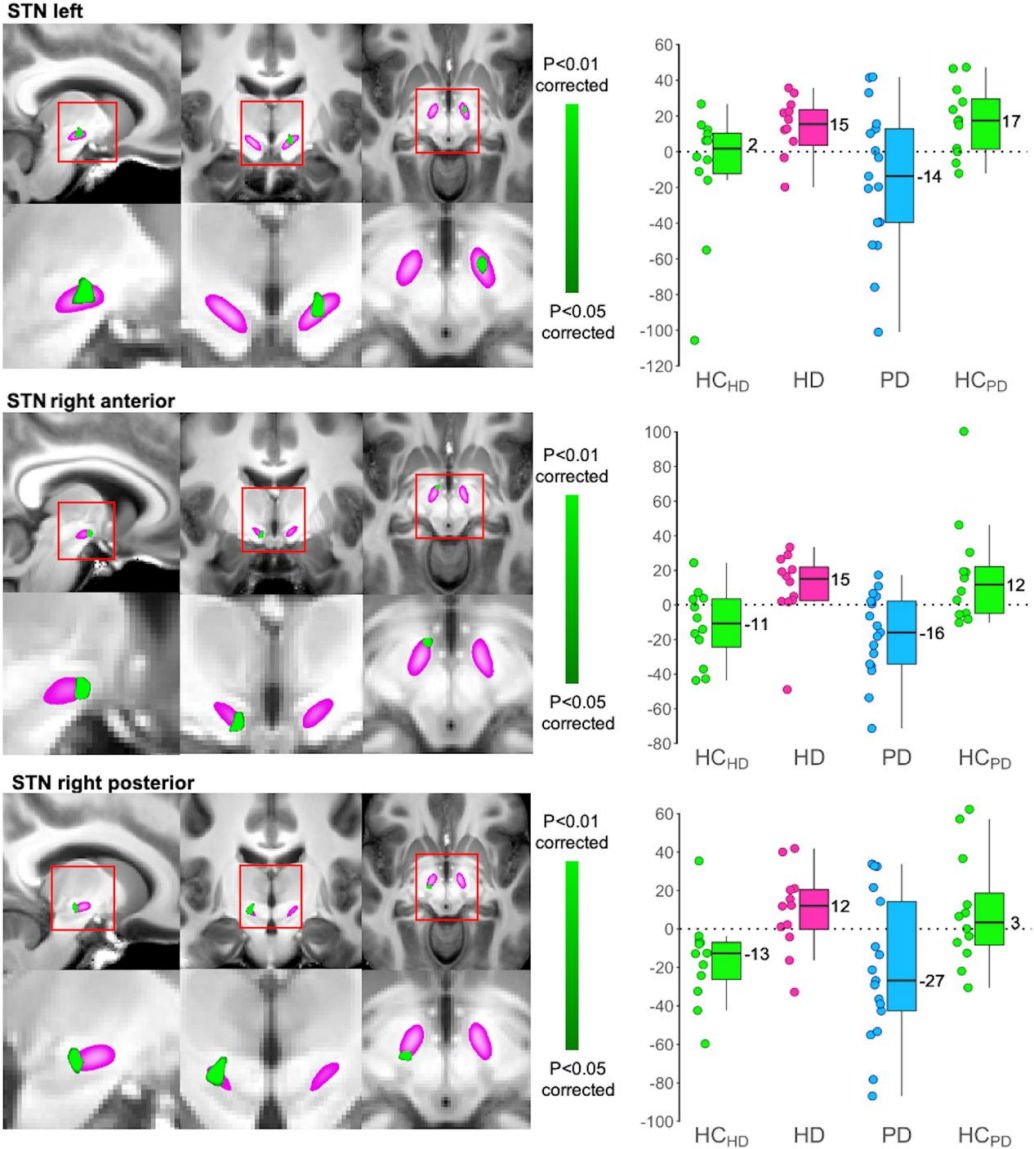
**Within the entire sensorimotor functional network (shown in Fig. 1), Huntington’s disease (HD) and Parkinson’s disease (PD) demonstrate opposite functional connectivity in the subthalamic nucleus (STN).** Left panel, three distinct clusters of significant (corrected using false discovery rate) group comparison results (in green) were found within the STN (in pink): the left STN, and the anterior and posterior regions of the right STN, the latter at the border with the substantia nigra pars reticulata (higher functional connectivity in HD compared with its corresponding matched healthy control group HC_HD_, lower functional connectivity in PD compared with its matched control group HC_PD_), based on multiple regressions and Student’s *t*-tests. For visualisation purposes clusters are shown at *P* < 0.05. Right panel, box plots based on functional connectivity values (parameter estimates) extracted from the significant STN clusters shown on the left (a.u.). Radiological orientation.

A formal statistical comparison between the four groups revealed significant *opposite* differences between HD and PD compared with their respective HC group in the STN (Fig. 2). We found that the HD participants showed *higher* functional connectivity of the STN within the sensorimotor network than the HC_HD_ group; conversely, the PD participants exhibited *lower* STN functional connectivity than the HC_PD_. We also found the same opposite differences between HD and PD at trend level in the SN (likely in the pars reticulata) and GPe (Supplementary Fig. 2). We found no difference in functional connectivity in the GPi.

**Figure 3:**
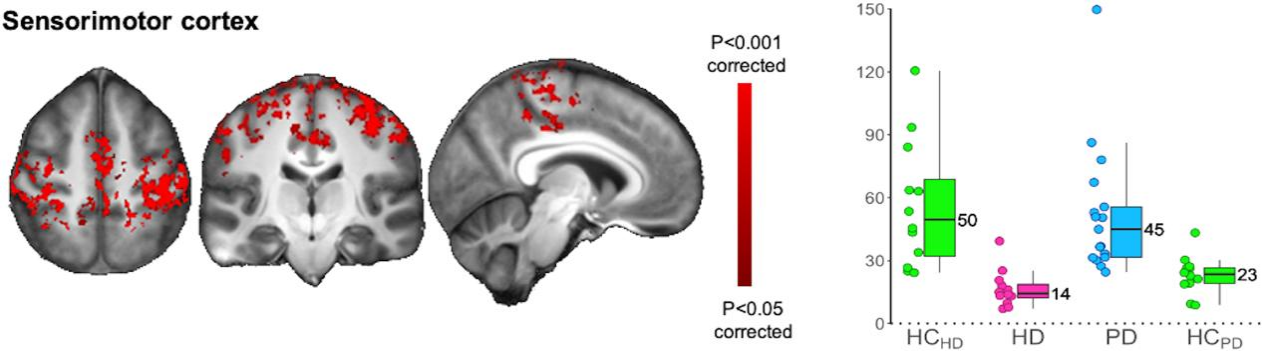
**Within the entire sensorimotor functional network (shown in Fig. 1), Huntington’s disease (HD) and Parkinson’s disease (PD) demonstrate opposite functional connectivity in the sensorimotor cortex.** Significant ANOVA group comparison results (in red) whereby HD and PD showed opposite differences (lower functional connectivity in HD compared with its corresponding matched healthy control group HC_HD_, higher functional connectivity in PD compared with its matched control group HC_PD_) from their respective control groups were found in the sensorimotor cortex region of interest. Left panel, significant (*P* < 0.05, corrected using threshold-free cluster enhancement) functional connectivity sensorimotor cortical results, based on multiple regressions and Student’s *t*-tests. Right panel, the corresponding box plots (parameter estimates, a.u.). Radiological orientation.

Within the sensorimotor cortex itself, visual comparison suggested overall a lower functional connectivity for HD carriers compared with HC_HD_; in contrast, PD patients seemed to show higher functional connectivity compared with HC_PD_, especially in the left hemisphere (Fig. 1B–E). This opposite functional connectivity within the sensorimotor cortex between HD and PD was formally confirmed by statistical comparison (Fig. 3).

Both sets of results, in the STN and in the sensorimotor cortex, remained virtually unchanged by adding sex as an additional confounder (Supplementary Fig. 3).

In addition, formal comparison between differences in left and right hemisphere for the same contrast revealed a trend for the effects being stronger in the left sensorimotor cortex than the right (lowest corrected *P*-value = 0.1, uncorrected *P*-value = 2 × 10^−4^).

Peak results, including local maxima MNI coordinates and effect sizes, are all reported in Supplementary Table 3.

### *Post hoc* age effect on sensorimotor functional connectivity in controls

The two HC groups, by construction, differ in age by 20 years on average to match the two patient groups. We formally tested *post hoc* whether age played a role in the HC on the functional connectivity values in the regions showing significant opposite effects between HD and PD. We found a significant effect of age, both within the sensorimotor cortex cluster (rho = −0.54, *P* = 0.006) and within the STN clusters (left: rho = 0.50, *P* = 0.013; right anterior: rho = 0.26, non-significant: *P* = 0.211; right posterior: rho = 0.43, *P* = 0.038).

### Correlations between rs-fMRI results and clinical scores

**Figure 4:**
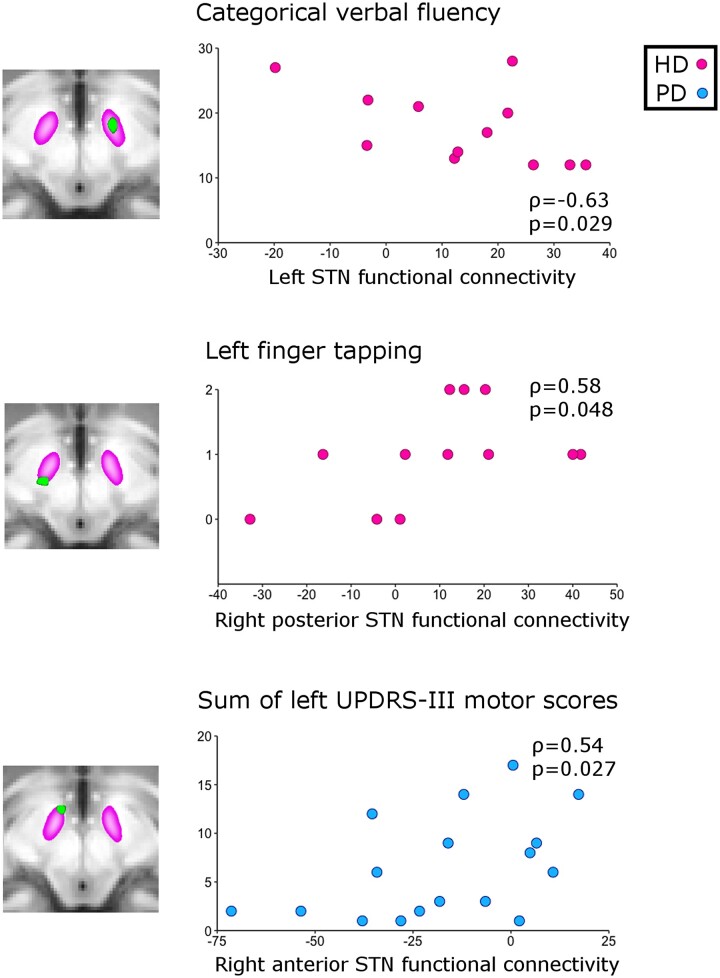
**Examples of correlation plots between significant functional connectivity values in each subthalamic nucleus (STN) cluster and clinical measures.** In pink, two top associations within the Huntington’s disease (HD) group: between left STN functional connectivity and categorical verbal fluency, and between right posterior STN functional connectivity and left hand finger tapping. In blue, one top association within the Parkinson’s disease (PD) group: between STN right anterior functional connectivity and the sum of all the left-lateralised motor scores in the unified Parkinson’s disease rating scale UPDRS-III, based on ρ (rho) values from Table 2 (Spearman’s rank correlation coefficient).

For the HD group, correlations between functional connectivity values in the clusters of interest and clinical scores consistently revealed associations related to the pathological effect of the disease, i.e. worse symptoms were associated with functional connectivity values furthest away from the values of HC (Table 2, Fig. 4).

**Table 2:** Correlations between functional connectivity results and clinical and behavioural measures for Huntington’s disease (HD) and Parkinson’s disease (PD) groups

HD carriers					
rs-fMRI		UHDRS/HLVT item	Spearman's correlation		
Group contrast	Brain region		Rho	*P*-value	FDR 0.1
(HD−HC_HD_)−(PD−HC_PD_)	STN (left)	Categorical verbal fluency test correct	−0.63	0.0292	
		Hopkins verbal learning test delayed recall	−0.61	0.0352	
	STN (right anterior)	CAG expansion repeats	−0.63	0.0395	
	STN (right posterior)	Finger taps, left	0.58	0.0479	
−(HD−HC_HD_)+(PD−HC_PD_)	Sensorimotor cortex	Disease-burden score	−0.88	0.0007	✓
		CAG expansion repeats	−0.80	0.0029	✓
		Luria	−0.78	0.0027	✓
		Tandem walking	−0.73	0.0068	✓
		Maximal chorea upper extremity, left	−0.71	0.0097	✓
		Retropulsion pull test	−0.71	0.0098	✓
		Diagnostic confidence score	−0.67	0.0178	
		Finger taps, left	−0.65	0.0225	
		Maximal dystonia trunk	−0.62	0.0326	
		Maximal chorea face	−0.62	0.0326	
		Total motor score	−0.61	0.0365	
		Saccade initiation, vertical	−0.59	0.0455	
		Maximal dystonia upper and lower extremities, left and right	−0.58	0.0467	
		Pronate/supinate hand, right	−0.58	0.0484	
		Hopkins verbal learning test 2	0.82	0.0010	✓
		Letter verbal fluency test S sub-total	0.73	0.0066	✓
		Stroop word reading correct	0.73	0.0069	✓
		Hopkins verbal learning test 3	0.70	0.0122	
		Hopkins verbal learning test 1	0.68	0.0147	
		Categorical verbal fluency test correct	0.66	0.0208	
		Hopkins verbal learning test delayed recall	0.65	0.0214	
		Stroop colour naming correct	0.65	0.0218	
		Symbol digit modalities test	0.67	0.0242	
		Stroop word reading errors	−0.64	0.0251	
		Categorical verbal fluency test perseverations	−0.59	0.0455	
		Functional occupation	0.62	0.0326	
		Functional assessment	0.61	0.0368	
		Independence scale	0.61	0.0368	

PD patients					
rs-fMRI		UPDRS item	Spearman's correlation		
Group contrast	Brain region		Rho	*P*-value	FDR 0.1
(HD−HC_HD_)−(PD−HC_PD_)	STN (right anterior)	III—Sum motor scores, left ^a^	0.54	0.0267	
		III—Asymmetry of symptoms	0.50	0.0392	
		I—Urinary problems	−0.52	0.0323	
	STN (right posterior)	Composite posture and gait b	−0.75	0.0006	✓
		II—Chewing and swallowing	−0.60	0.0106	
		III—Medicated for PD	−0.60	0.0111	
		I—Apathy	−0.50	0.0391	
− (HD−HC_HD_) + (PD−HC_PD_)	Sensorimotor cortex	Composite posture and gait	0.53	0.0289	
		I—Depressed mood	−0.63	0.0067	
		I—Anxious mood	−0.58	0.0147	

Correlation results between each supra-threshold resting-state functional MRI (rs-fMRI) result in the subthalamic nucleus (STN) and sensorimotor cortex, and clinical and cognitive measures. Scores are organised into motor, cognitive, and other items. Uncorrected *P*-values are reported; those surviving false discovery rate (FDR) correction at 10% across all the clinical and behavioural measures are marked with ✓. HC_HD_, healthy control group matched to the HD group; HC_PD_, healthy control group matched to the PD group; UHDRS, unified Huntington’s disease rating scale; HVLT, Hopkins verbal learning test; UPDRS, unified Parkinson’s disease rating scale.

^a^‘III—Sum motor score, left’ is the sum of all the left-lateralised motor scores in UPDRS-III.

^b^‘Composite posture and gait’ is the sum of UPDRS items involving posture and gait.

In particular, abnormal functional connectivity in the STN showed pathological association with delayed recall and verbal fluency, and with finger tapping for the posterior STN cluster. We also found numerous correlations within the HD group between the functional connectivity values in the sensorimotor cortex cluster and several motor, cognitive and functional items. The strongest pathological associations for motor scores were with the Luria test, and gait, chorea and postural instability symptoms. For the cognitive domain, the strongest correlations were found with immediate recall, verbal fluency and word naming, and there were moderate associations with functional scores. In addition, both CAG and/or disease burden were correlated with functional connectivity in the anterior STN, and in the sensorimotor cortex (with the highest association: rho = −0.88, with disease burden). Of note, the association between CAG and functional connectivity values in the anterior STN was the only correlation within the HD group compensatory in nature (i.e. in opposite direction to pathological).

In the PD patients, functional connectivity in the posterior STN cluster revealed consistent pathological association, most strongly with a composite measure of postural instability and gait, with dysarthria and dysphagia, as well as apathy, and whether the patients were taking medication (Table 2, Fig. 4). Functional connectivity in the right anterior STN cluster, on the other hand, showed a possibly compensatory association with the summary measure of UPDRS-III (left lateralised), but a pathological one with bladder function. Higher functional connectivity in this cluster was also associated with left-lateralised symptoms. Cortical sensorimotor functional connectivity values were correlated with items from both UPDRS-I, -II and -III sections. While the association with the composite measure of postural instability and gait seemed a pathological consequence of the disease—similarly to that observed with the posterior STN—the correlations with depression and anxiety went in the opposite direction, perhaps partly as a result of them being moderately negatively correlated with one another (e.g. depressed mood and composite measure of posture and gait: rho = −0.4).

For both HD and PD groups, additional correlations with the functional connectivity values in SN (likely in the pars reticulata) and GPe are reported in Supplementary Table 4.

## Discussion

Our study has revealed that HD and PD exhibit opposite pattern of functional connectivity of the STN and the sensorimotor cortex. Such an altered resting state of the STN is clinically meaningful, with the severity of deviation from controls largely associated with worsening motor and cognitive symptoms, despite functional connectivity going in opposite directions in each disorder. Similarly, we have also found an opposite pattern in the resting state of the sensorimotor cortex itself between HD and PD. These results take full advantage of the high resolution afforded by using a high-field 7T scanner to investigate the functional resting state of a small structure such as the STN, and from the fact that both participants with HD and PD were involved for the first time in the same MRI study.

State-of-the-art 7 T scanner and EPI sequence, using both GRAPPA and multi-band acceleration approaches, made it possible to achieve an isotropic resolution of 1.2 mm. In other words, each voxel represented a volume of 1.7 mm^3^—twice as small as the highest resolution achieved so far to investigate the human STN function using whole-brain fMRI^17^—thus offering 70+ voxels to cover the entire ∼130 mm^3^ volume of the STN.^15^ This allowed us to distinguish STN from SN *directly* in the imaging modality of interest at the individual level (Supplementary Figs 1 and 4), but also to control for the fact that the volume of the STN did not differ between the two movement disorders groups and their respective control groups (*P* = 0.439 for HD versus HC_HD_ and *P* = 0.937 for PD versus HC_PD_). We deliberately used ICA to ensure that the signal we observed was specifically that of the functional connectivity of the STN within the sensorimotor network *above and beyond* any other resting-state contributions of the STN. While functional images needed to be smoothed to create a group-ICA resting-state template, we made sure to use a relatively small Gaussian kernel of 3 mm—which guarantees that 75% of the signal in the STN originates from this structure itself, and not from other surrounding tissue, esp. that of the SN.^15^ Crucially, once we obtained our sensorimotor resting-state network template, we regressed it into the *unsmoothed* data.

This sensorimotor network template encompassed regions of the primary cortex, premotor cortex and primary and secondary somatosensory cortex covering the upper and lower limbs.^44^ Our findings reflecting a differential effect of HD and PD on the functional connectivity within this network recapitulated all of its cortical regions and markedly so in the left hemisphere, in premotor and primary sensorimotor areas corresponding to the right hand (Supplementary Table 3). In this instance, functional connectivity thus represented the way this part of the cortex is synchronized with itself, and what we detected was probably the sum output of all three cortico-subcortical–cortical loops: direct, indirect and hyperdirect. This perhaps explains why we identified correlations with the more ‘global’ measure of disease burden in HD, or with a composite measure of postural instability and gait in PD. HD carriers demonstrated the most notable difference, showing lower functional connectivity and no overlap but for one carrier with the values in the HC_HD_ group. This is consistent with ICA cortical results observed both at rest and during task in premanifest HD.^45-47^ Conversely, and in line with a few previous resting-state studies using both magnetoencephalography and fMRI,^48,49^ PD patients showed higher functional connectivity in the sensorimotor cortex compared with the HC_PD_ group.

We have identified for the first time *in vivo* a differential effect of HD and PD in the human STN. In particular, the clear contrast of functional connectivity between the two movement disorders was observed in the unique cluster identified in the left STN, whose coordinates (*x* = −10, *y* = −11, *z* = −7) precisely corresponded to those of the DBS ‘sweet spot’ (*x* = −10, *y* = −13, *z* = −7).^50^ This sweet spot was identified as the cluster of maximum overall efficacy for improvement in rigidity, bradykinesia and tremor in advanced PD patients. These differences in the overall synchronization of the spontaneous oscillations of the STN within the sensorimotor network—likely the sum of the contributions from the hyperdirect and indirect pathways—were clinically meaningful, and associated with worsening of the symptoms in both PD and HD. While the PD population included here presented with very early disease (89% with unilateral symptoms, 85% with H&Y stage I off medication) unlike the DBS study of Akram and colleagues,^50^ we nonetheless observed a trend between STN functional connectivity and rigidity of the upper extremities (rho = 0.45; *P* = 0.07). This area of the STN also encompasses associative connections,^16,51^ likely explaining the correlations observed between two cognitive tests (verbal fluency and HVLT delayed recall) and STN functional connectivity in the HD carriers (Table 2). Similarly, the functional connectivity of two other clusters showing opposite impact of the two movement disorders on the STN were associated with the worsening of motor symptoms, especially in the posterior cluster corresponding mainly to motor connections^16,51^: finger tapping in HD carriers, and postural instability and gait in PD patients (Table 2, Fig. 4). Conversely, the functional connectivity of the anterior STN—in a region typically mostly connected to prefrontal cortical areas, and only to a lesser extent to motor areas—seemed to show compensatory associations with CAG repeats in the HD group, and with UPDRS-III in the PD group^51^ (Table 2, Fig. 4). This is similar to what was observed in the striato-cortical connections in PD in a previous study,^52^ perhaps suggesting the remapping of motor connections within the STN towards more anterior regions, to compensate for those motor regions which may be the most affected by both diseases in the typically more posterior parts of the STN.

Changes in functional connectivity are notoriously hard to interpret,^53,54^ and DBS electrophysiology unfortunately cannot inform the interpretation of such slow oscillations as those investigated in rs-fMRI (0.01–0.1 Hz).^55^ One possibility is that the changes in functional connectivity observed here in PD and HD reflect an opposite shift in the synchronization between sensorimotor cortex and STN signals,^54^ as a result of the pathological alteration in the influence of indirect and hyperdirect pathways. Importantly, it also has been reported recently that an imbalance in excitation:inhibition (E:I) ratio, such as precisely witnessed in PD and HD, could be related to functional connectivity in the same regions.^56^ Indeed, an *increase* in the E:I ratio appears to cause an increase in neuronal firing and relative blood flow, but remarkably a *reduction* in local and long-range functional connectivity in the same brain regions.^56^ Our results showing a decrease in functional connectivity in the sensorimotor cortex are thus in line with the increase of excitation that is known to happen in this region in HD; and vice versa in PD. Similarly, the findings of a reduced functional connectivity in the STN of PD patients tie in with the known overall increase in the E:I balance caused by a decrease of the inhibitory input onto the STN; and vice versa in HD. More specifically, in HD, pathological changes resulting in a vastly decreased inhibitory indirect pathway (possibly together with a modestly increased excitatory hyperdirect pathway), lead overall to the reduced excitation in the net output of the STN, and so a decrease in the E:I balance.^57^ Relatively recent animal models studies of HD seem to indeed confirm a reduced activity of the STN.^11^ For the same reasons, the fact that the STN is at the centre of convergence between an excitatory and an inhibitory pathway also probably explains why, on balance, there is on average no functional connectivity at rest of the STN within the sensorimotor network in HC, contrary to what we can be observed separately in the HD and the PD groups (Fig. 2).

A noticeable difference in functional connectivity, not only in the STN, but also in the sensorimotor cortex itself, can be also observed *between* the two healthy groups, which differ by 20 years to match the HD and PD groups. We thus tested for a possible age-related effect on these sensorimotor cortical and STN functional connectivity values in the HC, and indeed found a significant impact of age in both regions. By construction our main inference model, contrasting both patient groups with their respective matched control groups to account more strictly for age differences than by simply adding age as a covariate of no interest, makes it easier to—but by no means limited to—detect differences between HD and PD that go in opposite direction to differences between the two healthy groups. This produced the additional effect of uncovering an intriguing altering of the sensorimotor functional connectivity, both in the sensorimotor cortex itself, and in the STN, with healthy ageing. This result is in line with a previous study that showed an increased functional connectivity with age between the STN and sensorimotor cortex,^58^ as well as with a recent study demonstrating that a decrease of functional connectivity with age in the sensorimotor cortex is due to a decrease in inhibition—and thus an increase in the E:I balance.^59^

A clear limitation of this study is that resting-state functional connectivity does not allow us to distinguish between the contribution of the indirect and the hyperdirect pathways to the STN, unlike effective functional connectivity using DBS recordings^60^ (although very recent methodological developments suggest this might be possible with a sufficiently fast sampling rate^61^). However, this can also be considered as a strength in that what we observe here, non-invasively and in participants with very early disease, is the reflection of the *overall* effect of each of the two movement disorders on the functional connections of the sensorimotor cortex and the STN. Another strength of rs-fMRI is that, while task-activation studies have a poor signal-to-noise ratio because the signal (task-related modulation) is often small relative to the ongoing ‘noise’ and accounts for at best about 20% of the blood oxygenation level dependent (BOLD) variance, most of this ‘noise’ is actually made of spontaneous activity which is precisely the focus of rs-fMRI and can account for up to 80% of the variance, making rs-fMRI a potentially richer and more sensitive source of disease-related signal changes.^62^ Movement in the scanner can also be a possible confounding factor.^63^ We motion-corrected our imaging data,^31^ but also took great care in denoising every single rs-fMRI timeseries using single-subject ICA, in particular by regressing out from the data motion-related components.^33-35^ In addition, while the vast majority of our participants were either at a premanifest stage of HD, or at a very early unilateral stage of PD, we made sure that the one manifest HD carrier, and the three PD participants with H&Y two off medication—of which two were identified as having bilateral symptoms—did not drive our results (Supplementary Fig. 5). Similarly, the three unmedicated PD participants did not seem to exhibit any distinct pattern in their functional connectivity, except perhaps in the posterior part of the right STN, which likely explains the significant correlation observed in this specific cluster and medication status from the UPDRS-III (Supplementary Fig. 6).

This work’s most obvious limitation resides in the inherently low number of participants included, particularly for the two clinical populations HD and PD. We note however that, as long as valid statistical testing is used—in our case, using non-parametric testing and focusing on supra-threshold clusters fully corrected for multiple comparisons over space—the actual effect observed has to be larger for a small group to reach significance than it would have needed to be for a larger group.^64^ More importantly, this MRI study is unique in that it not only combines for the first time HD and PD participants—both at a very early stage—but in that it leverages the high signal-to-noise offered by the 7T scanner to achieve the highest resolution of any resting-state study in these populations and of the STN. This made it possible to explore the complex interplay between the disease effects and their overall contribution on the STN, demonstrating for the first time *in vivo* in humans a differential, clinically meaningful effect of each movement disorder on the sensorimotor cortex and STN.

## Supplementary Material

fcad282_Supplementary_Data

## Data Availability

All manually delineated ROIs, whole-brain functional connectivity dual-regression sensorimotor maps, and statistical designs are freely available on this dedicated webpage: https://open.win.ox.ac.uk/pages/douaud/hd-pd-smn/.
