# Supplementary material for: Subthalamic nucleus shows opposite functional connectivity pattern in Huntington’s and Parkinson’s disease
Source: Brain Commun. 2023 Dec 6;5(6):fcad282. doi: 10.1093/braincomms/fcad282 (PMC10699743; doi:10.1093/braincomms/fcad282)
Supplement: fcad282_Supplementary_Data [file fcad282_supplementary_data.pdf]

Supplementary Material

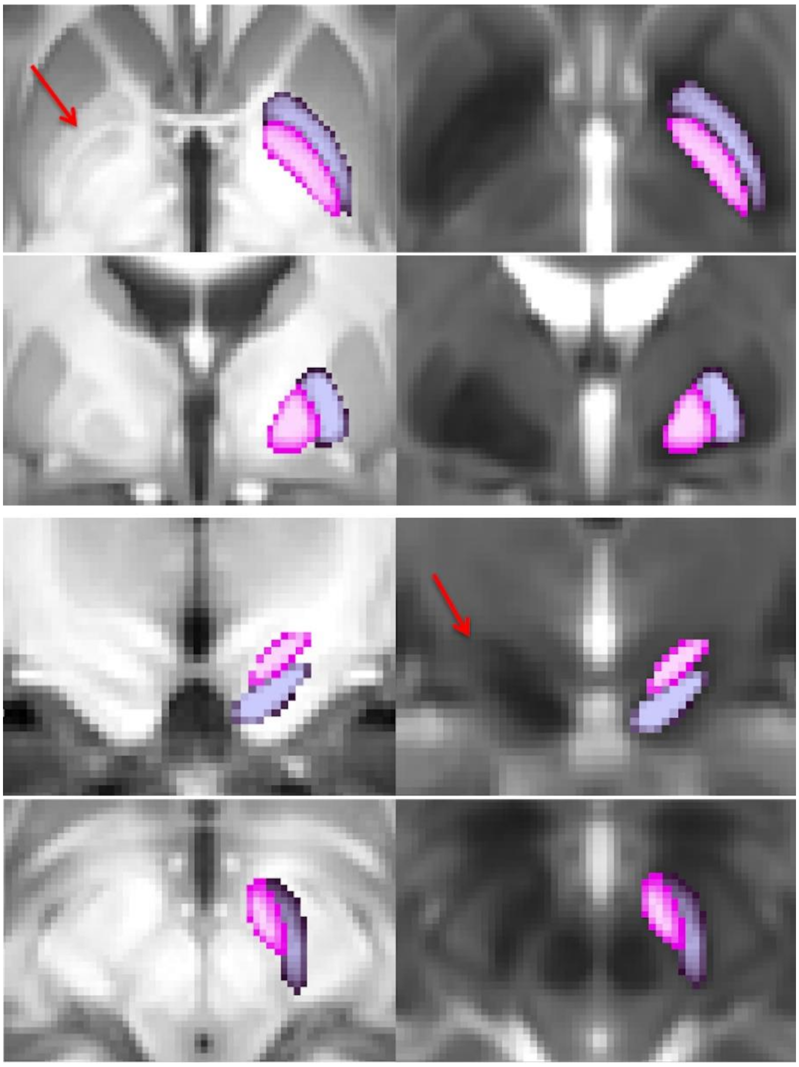

Supplementary Figure 1: Probabilistic maps of manually-drawn regions of interest (ROIs), averaged across all participants. These probabilistic ROIs are overlaid on the average structural T1 over proton density map (T1/PD) across subjects (left), and functional images averaged across all 220 volumes and across subjects (right). Of note, these background images, despite being averaged across *all* participants, still display very crisp contours. This highlights the clear benefit of our optimised registration strategy. Individual masks for the subthalamic nucleus (STN) can be seen in **Supplementary Figure 4**. Arrows point out the medullar lamina separating the internal globus pallidus (GPi) from the external globus pallidus (GPe) (ROI in pink and purple, respectively, top row), and the butterfly shape that makes it possible to distinguish STN from substantia nigra (SN) in the functional data (pink and purple, respectively, bottom row).

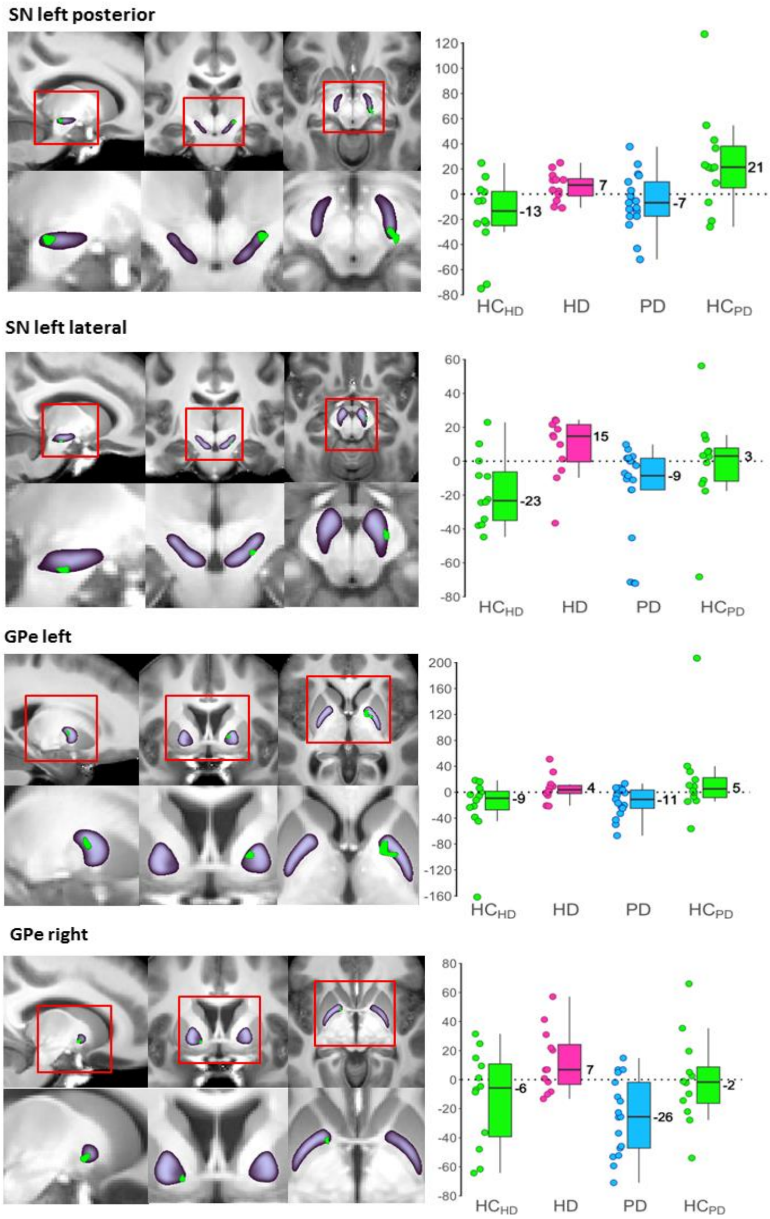

Supplementary Figure 2: Huntington's disease (HD) and Parkinson's disease (PD) demonstrate opposite functional connectivity in the substantia nigra (SN) and external globus pallidus (GPe). **Left**, Differential effects between HD and PD (green) were found in the left SN (purple), very likely the pars reticulata of the SN (SNr) (posterior SN, top; lateral SN, bottom). Based on multiple regressions and Student's t-tests. **Right**, differential effects (green) in the GPe (purple)(anterior left, top; anterior right, bottom). For visualisation purposes clusters are shown at $p < 0.05$, but those differences survived a more stringent uncorrected threshold of $p < 0.001$. Next to each brain views are the corresponding box plots based on values extracted from the SN and GPe supra-threshold clusters. Radiological orientation (left is right).

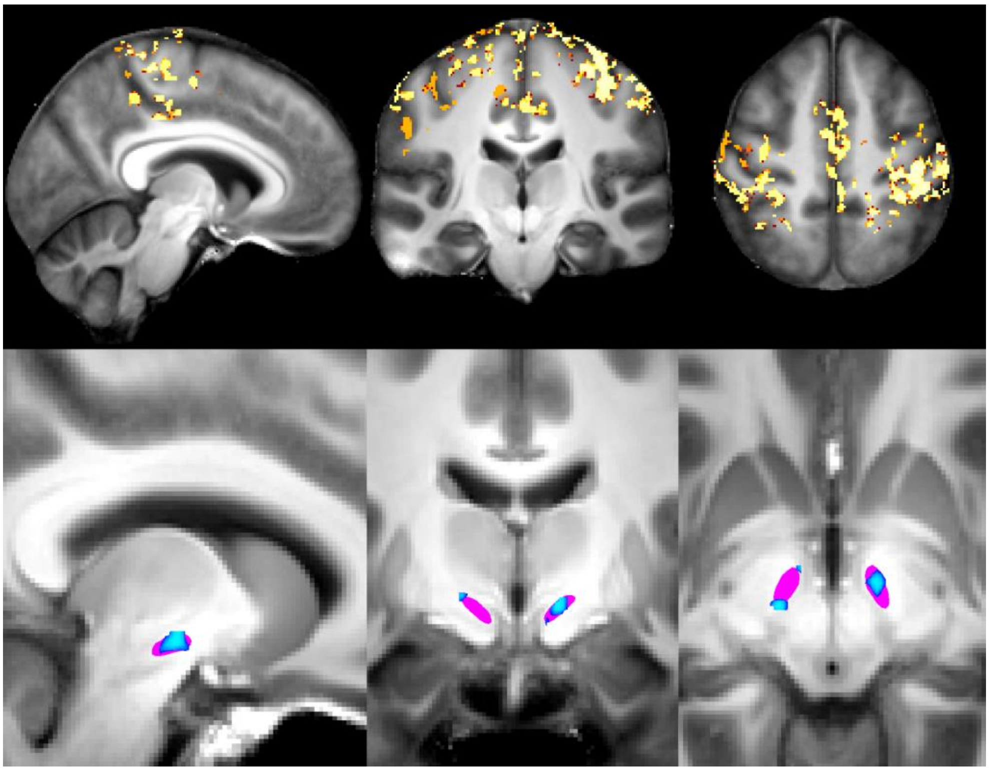

Supplementary Figure 3: Results showing opposite functional connectivity between Huntington's disease (HD) and Parkinson's disease (PD) after adding sex as a confounding covariate remain largely unchanged. Top, within the sensorimotor cortex ($p < 0.05$, corrected using threshold-free cluster enhancement). **Bottom**, in the subthalamic nucleus (STN, mask in pink) (for visualisation purposes, clusters are shown in blue at $p < 0.05$). Radiological orientation (left is right). Based on multiple regressions and Student's t-tests.

STN left

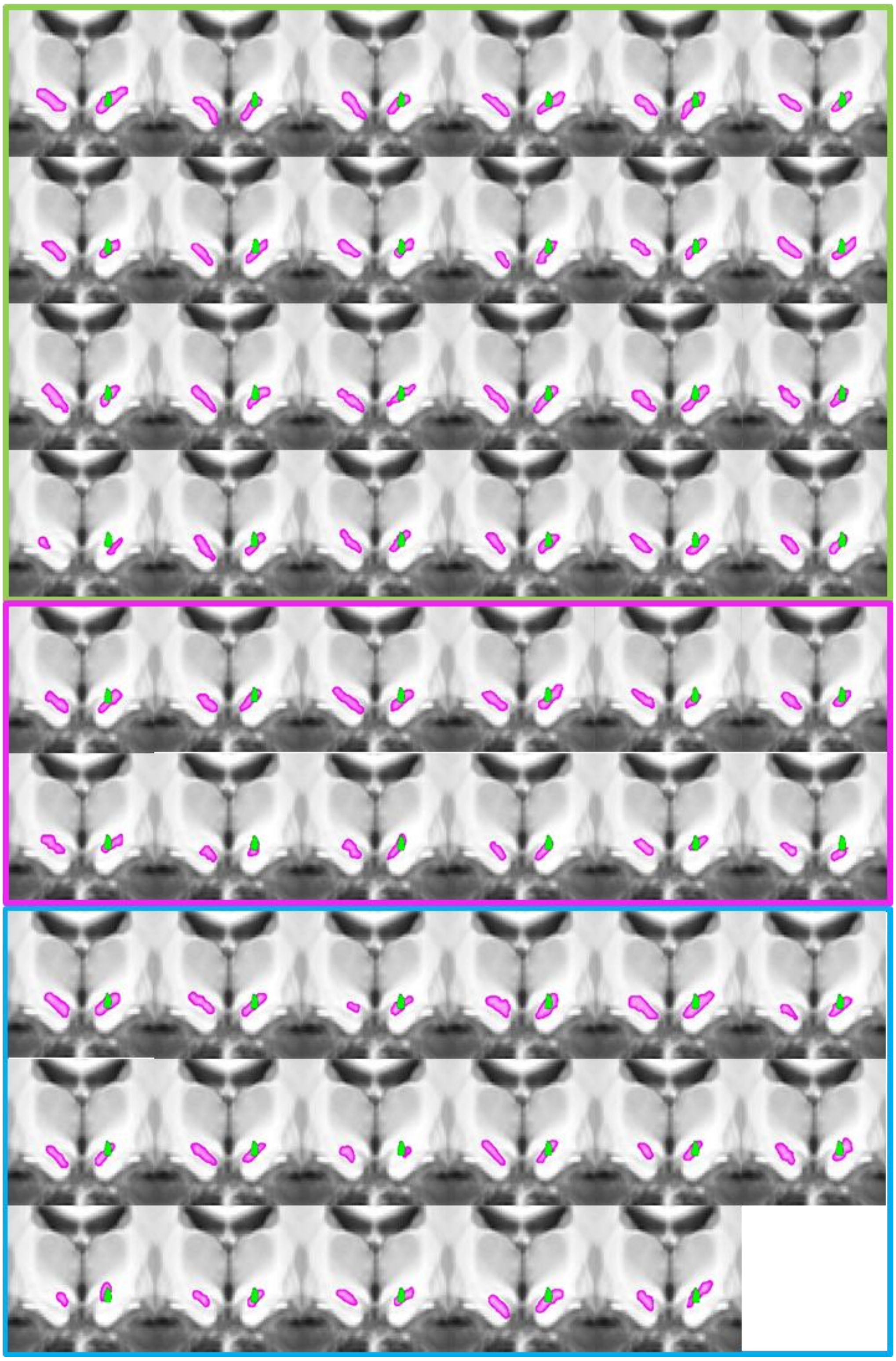

STN right anterior

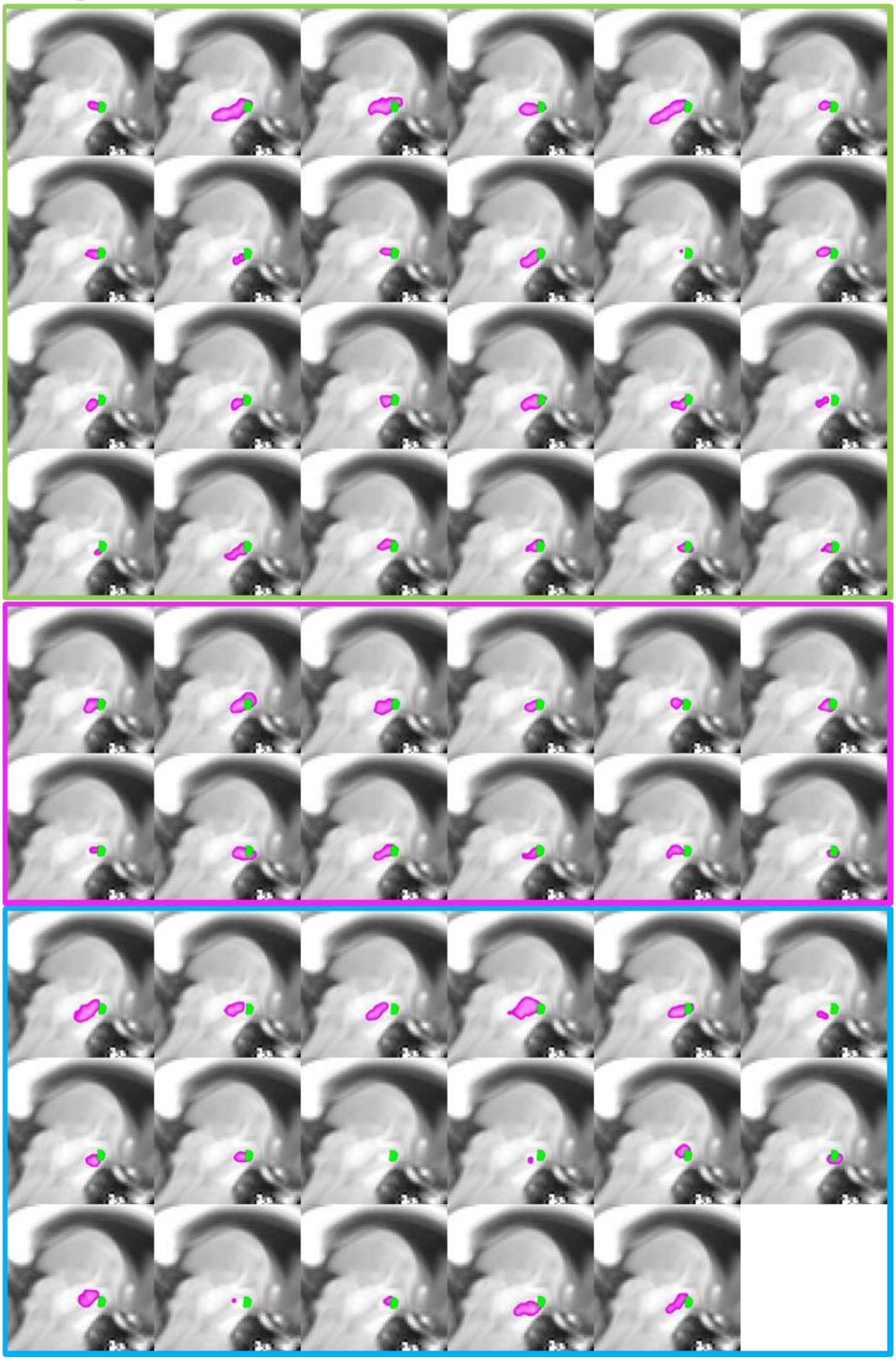

STN right posterior

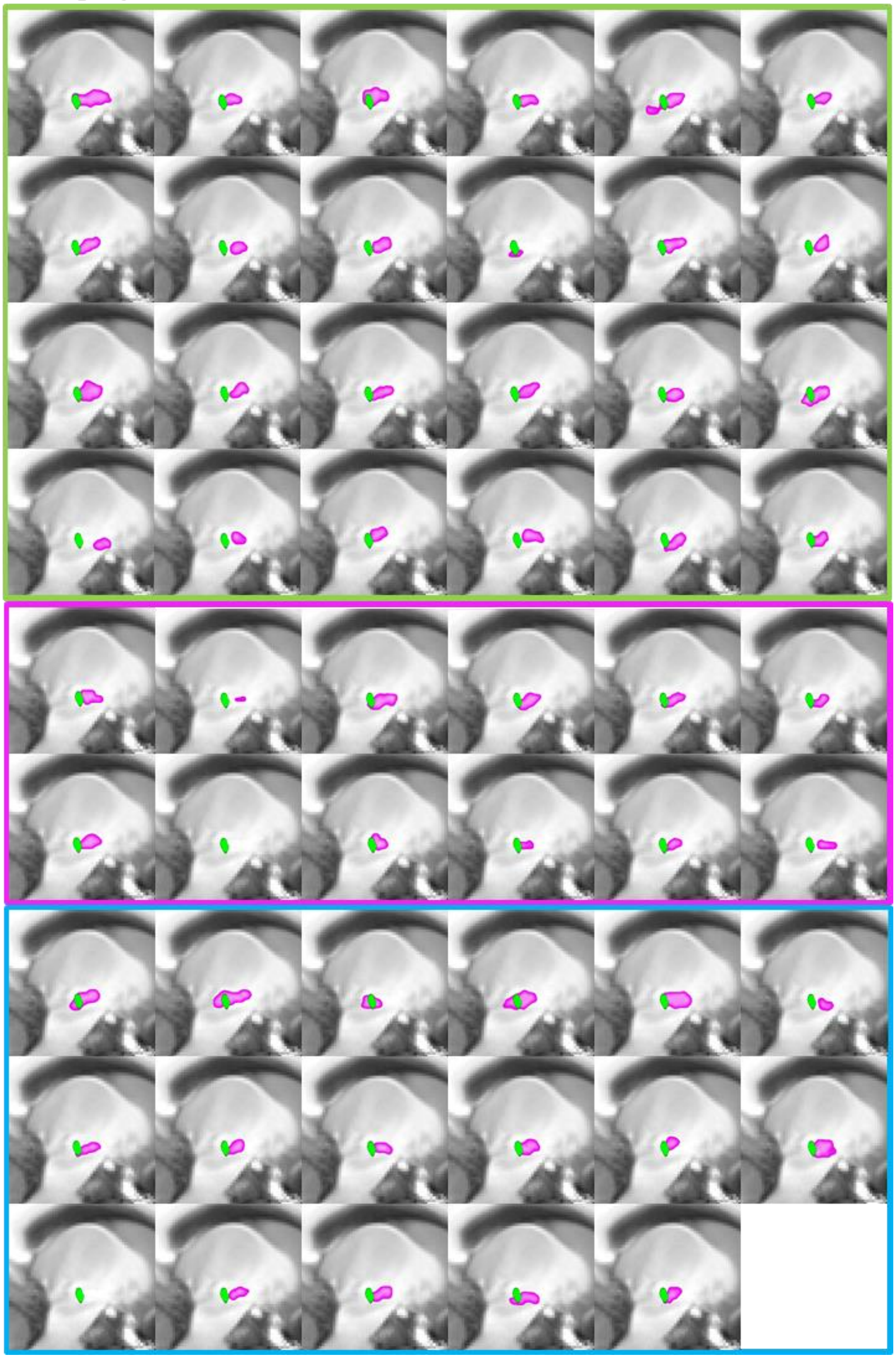

Supplementary Figure 4: Subthalamic nucleus (STN) results overlaid onto every individual mask of the structure. Significant clusters (green) are shown overlaid onto each individual mask for the STN (pink). For visualisation purposes clusters are shown at $p < 0.05$. Images for each healthy control, Huntington's disease, and Parkinson's disease group are respectively bordered in green, pink and blue boxes. Radiological orientation (left is right).

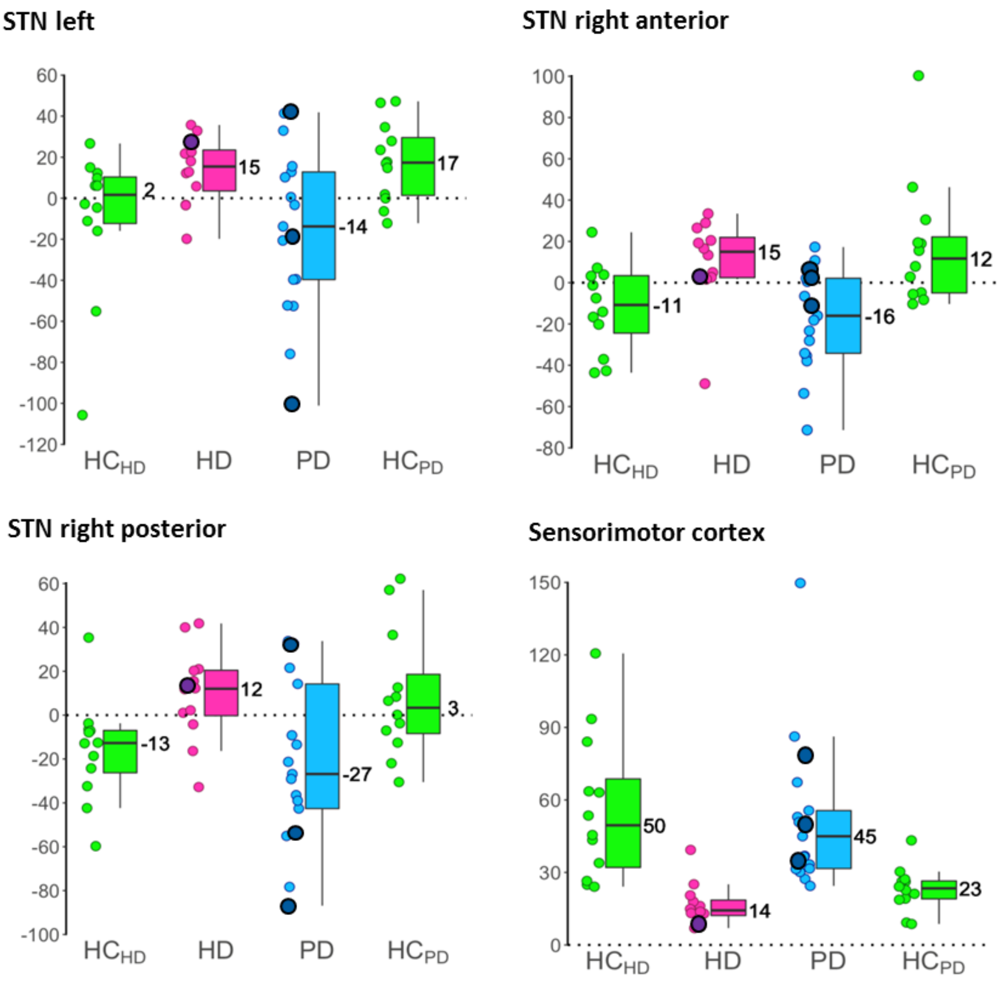

Supplementary Figure 5: Box plots of the main subthalamic nucleus (STN) and sensorimotor cortical results (Figures 2 and 3) highlighting the one manifest Huntington's disease (HD) carrier, and the three Parkinson's disease (PD) participants with a Hoehn and Yahr of 2 off medication.

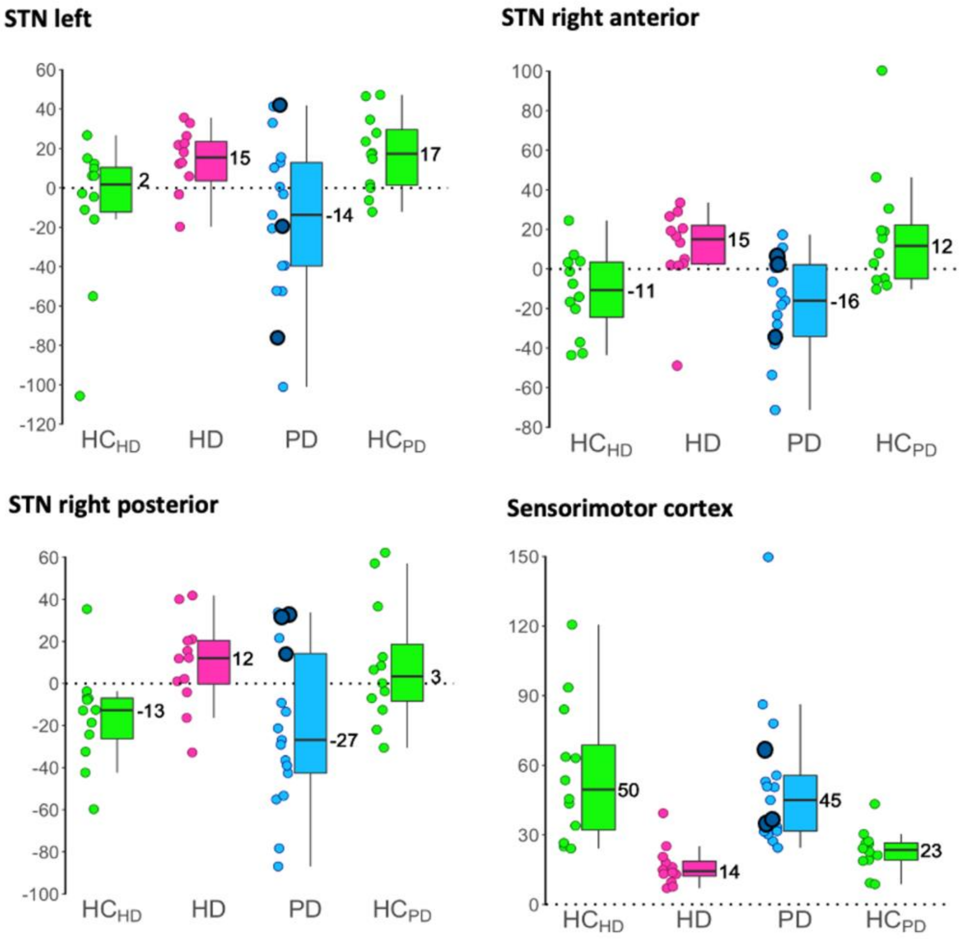

Supplementary Figure 6: Box plots of the main subthalamic nucleus (STN) and sensorimotor cortical results (Figures 2 and 3) highlighting the three unmedicated Parkinson's disease (PD) participants.

Supplementary Table 1: Detailed clinical measures for Huntington's disease (HD) carriers and Parkinson's disease (PD) patients. HD table abbreviations: LVFT = lexical verbal fluency test. CVFT = categorical verbal fluency test. SDMT = symbol digit modalities test. HVLT = Hopkins verbal learning test. PD table abbreviations: TD = tremor dominant. PIGD = postural instability and gait disturbance. ID = indeterminate. H&Y = Hoehn and Yahr.

HD carriers				
**Group**		**All**	**Premanifest**	**Manifest**
**N**		12	11 (92%)	1 (8%)
**UHDRS - Motor assessment**				
	Ocular pursuit	0.2 ±0.4 (0 - 1)	0.2 ±0.4 (0 - 1)	0
	Saccade initiation	0.5 ±1.0 (0 - 3)	0.4 ±0.9 (0 - 3)	2
	Saccade velocity	0.4 ±0.7 (0 - 2)	0.3 ±0.5 (0 - 1)	2
	Tongue protrusion	0	0	2
	Finger taps	1.8 ±1.3 (0 - 4)	1.6 ±1.2 (0 - 4)	4
	Pronate/supinate hands	1.5 ±1.2 (0 - 3)	1.4 ±1.1 (0 - 3)	3
	Luria	0.4 ±0.7 (0 - 2)	0.3 ±0.5 (0 - 1)	2
	Rigidity arms	0.8 ±1.1 (0 - 4)	0.7 ±1.2 (0 - 4)	1
	Bradykinesia-body	0.4 ±0.7 (0 - 2)	0.5 ±0.7 (0 - 2)	0
	Maximal dystonia	1.1 ±2.1 (0 - 6)	1.0 ±2.2 (0 - 6)	2
	Maximal chorea	1.9 ±3.0 (0 - 9)	1.5 ±2.8 (0 - 9)	6
	Gait	0.4 ±0.7 (0 - 2)	0.5 ±0.7 (0 - 2)	0
	Tandem walking	0.3 ±0.7 (0 - 2)	0.4 ±0.7 (0 - 2)	0
	Retropulsion pull test	0.3 ±0.7 (0 - 2)	0.3 ±0.6 (0 - 2)	1
**UHDRS - Functional capacity**				
	Occupation	2.6 ±0.9 (0 - 3)	2.8 ±0.4 (2 - 3)	0
	Finances	2.5 ±1.0 (0 - 3)	2.6 ±0.9 (0 - 3)	1
	Domestic chores	1.9 ±0.3 (1 - 2)	2	1
	Activities of daily living	2.9 ±0.3 (2 - 3)	3	2
	Care level	2	2	2
**UHDRS - Functional assessment scale**		24.3 ±1.8 (19 - 25)	24.7 ±0.6 (23 - 25)	19
**UHDRS - Independence scale**		95.4 ±9.9 (70 - 100)	97.7 ±6.1 (80 - 100)	70
**UHDRS - Cognitive assessment**				
	LVFT-f	10.0 ±4.9 (2 - 18)	10.7 ±4.5 (4 - 18)	2
	LVFT-a	9.5 ±4.2 (5 - 20)	9.7 ±4.4 (5 - 20)	7
	LVFT-s	11.8 ±4.3 (5 - 19)	12.5 ±3.9 (7 - 19)	5
	Stroop color naming	76.3 ±25.9 (39 - 100)	79.0 ±25.2 (39 - 100)	46
	Stroop word reading	84.4 ±18.7 (54 - 100)	85.4 ±19.3 (54 - 100)	74
	Stroop interference	60.8 ±34.2 (23 - 100)	63.4 ±34.6 (23 - 100)	32
	ECVFT	17.8 ±5.8 (12 - 28)	18.3 ±5.8 (12 - 28)	12
	SDMT	46.4 ±13.4 (23 - 64)	48.7 ±11.5 (32 - 64)	23
	HVLT - delayed recall	8.7 ±2.4 (3 - 12)	9.2 ±1.7 (6 - 12)	3
**UHDRS - Behaviour assessment**				
	Depressed mood	0.4 ±1.2 (0 - 4)	0.4 ±1.2 (0 - 4)	1
	Anxiety	0.8 ±1.1 (0 - 3)	0.7 ±1.1 (0 - 3)	1
	Irritability	0.6 ±0.8 (0 - 2)	0.5 ±0.8 (0 - 2)	1
	Angry/aggressive behaviour	0.1 ±0.3 (0 - 1)	0	1
	Apathy	0.7 ±1.2 (0 - 3)	0.7 ±1.2 (0 - 3)	0
	Perseverative behaviour	0.4 ±0.7 (0 - 2)	0.4 ±0.7 (0 - 2)	1
	Disoriented behaviour	0.1 ±0.4 (0 - 1)	0	1

Following amended criteria described in the TRACK-HD study (Tabrizi et al., 2012, 2013), we classified the HD carriers based on a combination of total motor score (TMS), total functional capacity (TFC) and diagnostic confidence score (DCS). Eleven participants were at a premanifest stage (either early: DCS<4, TMS ≤ 5 and TFC=13; or late: DCS < 4 and either TMS > 5 or TFC < 13), and 1 participant showed manifest HD (DCS = 4). HVLT is actually not part of the UHDRS, but based on the TRACK-HD study; it was added to include a measure of delayed recall.

PD patients				
**Group**		**All**	**H&Y stage 1**	**H&Y stage 2**
**N**		18	15 (83%)	3 (17%)
**Disease Duration (months from diagnosis)**		33.1 ± 27.4 (1 - 89)	35.3 ± 26.8 (5 - 89)	22.0 ± 33.8 (1 - 61)
**Medicated for PD:**	**Yes**	15 (83%)	13 (72%)	2 (11%)
	**No**	3 (17%)	2 (11%)	1 (5.5%)
**Symptom Laterality**	**Unilateral**	16 (89%)	16 (89%)	0
	**Bilateral**	2 (11%)	0	2 (11%)
**PD Subtype:**	**TD**	16 (89%)	13 (72%)	3 (17%)
	**PIGD**	1 (5.5%)	1 (5.5%)	0
	**ID**	1 (5.5%)	1 (5.5%)	0
**I - Cognitive impairment**		0.11 ± 0.32 (0 - 1)	0.13 ± 0.35 (0 - 1)	0
**I - Depressed mood**		0.17 ± 0.38 (0 - 1)	0.20 ± 0.41 (0 - 1)	0
**I - Anxious mood**		0.28 ± 0.46 (0 - 1)	0.33 ± 0.49 (0 - 1)	0
**I - Apathy**		0.17 ± 0.38 (0 - 1)	0.13 ± 0.35 (0 - 1)	0.33 ± 0.58 (0 - 1)
**I - Sleep problems**		0.94 ± 1.00 (0 - 3)	0.87 ± 0.99 (0 - 3)	1.33 ± 1.15 (0 - 2)
**I - Daytime sleepiness**		0.67 ± 0.77 (0 - 2)	0.80 ± 0.77 (0 - 2)	0
**I - Pain and other sensations**		0.61 ± 0.70 (0 - 2)	0.67 ± 0.72 (0 - 2)	0.33 ± 0.58 (0 - 1)
**I - Urinary problems**		0.50 ± 0.62 (0 - 2)	0.53 ± 0.64 (0 - 2)	0.33 ± 0.58 (0 - 1)
**I - Constipation problems**		0.39 ± 0.78 (0 - 3)	0.47 ± 0.83 (0 - 3)	0
**I - Light headedness on standing**		0.22 ± 0.43 (0 - 1)	0.20 ± 0.41 (0 - 1)	0.33 ± 0.58 (0 - 1)
**I - Fatigue**		0.61 ± 0.61 (0 - 2)	0.60 ± 0.63 (0 - 1)	0.67 ± 0.58 (0 - 1)
**II - Speech**		0.61 ± 0.70 (0 - 2)	0.60 ± 0.74 (0 - 2)	0.67 ± 0.58 (0 - 1)
**II - Saliva and drooling**		1.00 ± 1.14 (0 - 3)	0.93 ± 1.10 (0 - 3)	1.33 ± 1.53 (0 - 3)
**II - Chewing and swallowing**		0.33 ± 0.77 (0 - 3)	0.20 ± 0.41 (0 - 1)	1.00 ± 1.73 (0 - 3)
**II - Eating tasks**		0.33 ± 0.59 (0 - 2)	0.27 ± 0.46 (0 - 1)	0.67 ± 1.15 (0 - 2)
**II - Dressing**		0.33 ± 0.49 (0 - 1)	0.27 ± 0.46 (0 - 1)	0.67 ± 0.58 (0 - 1)
**II - Hygiene**		0.22 ± 0.43 (0 - 1)	0.20 ± 0.41 (0 - 1)	0.33 ± 0.58 (0 - 1)
**II - Handwriting**		1.00 ± 0.91 (0 - 3)	1.07 ± 0.96 (0 - 3)	0.67 ± 0.58 (0 - 1)
**II - Doing hobbies and other activities**		0.28 ± 0.46 (0 - 1)	0.27 ± 0.46 (0 - 1)	0.33 ± 0.58 (0 - 1)
**II - Turning in bed**		0.17 ± 0.38 (0 - 1)	0.13 ± 0.35 (0 - 1)	0.33 ± 0.58 (0 - 1)
**II - Getting out of bed/car/deepchair**		0.33 ± 0.59 (0 - 2)	0.20 ± 0.41 (0 - 1)	1.00 ± 1.00 (0 - 2)
**III - On levodopa**		0.50 ± 0.51 (0 - 2)	0.47 ± 0.52 (0 - 1)	0.67 ± 0.58 (0 - 1)
**III - Minutes since last dose**		861 ± 90 (740 - 980)	869 ± 98 (740 - 980)	833 ± 74 (780 - 885)
**III - Asymmetry of symtoms**		−0.40 ± 0.91 (−1 - 1)	−0.20 ± 1.01 (−1 - 1)	0 ± 1 (−1 - 1)
**Composite tremor**		6.61 ± 4.43 (0 - 14)	6.00 ± 3.89 (0 - 13)	9.67 ± 6.66 (2 - 14)
**Composite posture and gait**		0.44 ± 0.51 (0 - 1)	0.40 ± 0.51 (0 - 1)	0.67 ± 0.58 (0 - 1)
**Composite rigidity**		2.94 ± 1.21 (1 - 6)	2.93 ± 1.16 (1 - 6)	3.00 ± 1.73 (2 - 5)
**III - Sum motor scores , right**		7.94 ± 4.96 (0 - 16)	7.47 ± 4.98 (0 - 16)	10.3 ± 5.03 (5 - 15)
**III - Sum motor scores, left**		6.38 ± 5.12 (1 - 17)	5.60 ± 5.14 (1 - 17)	10.33 ± 3.21 (8 - 14)
**III - Sum motor scores, axial**		4.28 ± 2.59 (0 - 10)	3.87 ± 2.64 (0 - 10)	6.33 ± 0.58 (6 - 7)
**IV - Hours asleep**		7.09 ± 0.99 (5 - 8.5)	7.21 ± 0.87 (5.5 - 8.5)	6.50 ± 1.50 (5 - 8)
**IV - Hours awake**		16.9 ± 0.99 (15.5 - 19)	16.8 ± 0.87 (15.5 - 18.5)	17.5 ± 1.5 (16 - 19)
**IV - Time spent with dyskinesias**		0.11 ± 0.32 (0 - 1)	0.07 ± 0.26 (0 - 1)	0.33 ± 0.58 (0 - 1)
**IV - Time spent in the off state**		0.22 ± 0.43 (0 - 1)	0.27 ± 0.46 (0 - 1)	0
**IV - Functional impact of fluctuations**		0.39 ± 0.98 (0 - 3)	0.47 ± 1.06 (0 - 3)	0
**IV - Complexity of motor fluctuations**		0.44 ± 1.04 (0 - 4)	0.53 ± 1.13 (0 - 4)	0

Measures are reported as average ± standard deviation (range).
Items that are null for all subjects (specifically: dysarthria, suicidal ideation, obsessive-compulsive behavior, delusions/paranoid thinking and hallucinations for HD carriers; I – Hallucinations and psychosis, I – Features of DDS, II – Freezing, III - Freezing of gait, III - Dyskinesia present during examination, IV – Functional impact of dyskinesia, IV – Painful off state dystonia for PD patients) are not reported in the table. UPDRS items that are included in the composite scores are not reported individually.

Supplementary Table 2. Summary demographic and clinical measures after exclusion of one healthy control (HC) and one Huntington's disease (HD) participant. Parkinson's disease (PD), HC after splitting into groups matched to HD and PD: HC_{HD} and HC_{PD}. UHDRS = unified Huntington's disease rating scale; UPDRS = unified Parkinson's disease rating scale; H&Y = Hoehn and Yahr.

HD carriers			PD patients		
**N**		12	**N**		17
**Premanifest**		11 (92%)	**Disease Duration (months from diagnosis)**		32.5 ± 28.1 (1 - 89)
**Manifest**		1 (8%)	**H&Y (Off)**		1.18 ± 0.39 (1 - 2)
**CAG expansion repeats ***		42.2 ± 2.7 (37 - 46)		**Stage 1**	14 (82%)
**Disease-burden score ***		250.7 ± 119.0 (87 - 413)		**Stage 2**	3 (18%)
**UHDRS :**	**Total Motor Score**	10.3 ± 10.5 (1 - 27)	**UPDRS III (Off)**		20.1 ± 8.2 (9 -39)
	**Total Functional Capacity**	11.9 ± 2.2 (6 - 13)	**Medicated for PD *****	**Yes**	14 (82%)
	**Diagnostic Confidence**	1.5 ± 1.2 (0 - 4)		**No**	3 (18%)
	**Total Behavioural Score ****	3.0 ± 4.0 (0 - 14)	**Symptom Laterality**	**Unilateral**	15 (88%)
	**Total Cognitive Score ****	321.7 ± 95.6 (199 - 439)		**Bilateral**	2 (12%)

Demographics

Group	HC	HC_{HD}	HC_{PD}	HD	PD
**N**	24	12	12	12	17
**Gender (M/F)**	15 (63%) / 9 (38%)	7 (58%) / 5 (42%)	8 (67%) / 4 (33%)	5 (42%) / 7 (58%)	12 (71%) / 5 (29%)
**Age (Years)**	45.4 ± 17.0 (21 - 77)	35.6 ± 14.7 (21 - 69)	55.3 ± 13.4 (29 - 77)	39.8 ± 11.4	59.2 ± 8.3 (47 - 72)
**Handedness**	22 R / 2 L	10 R / 2 L	12 R	8 R / 4 L	13 R / 4 AMB

Supplementary Table 3: Montréal Neurological Institute (MNI) coordinates and size effects for the local maxima of supra-threshold resting-state functional MRI results from group comparisons. In the cortex, these were identified using both the Jülich probabilistic atlas (Eickhoff et al., 2005) and using the somatotopic maps kindly provided by (Zeharia et al., 2015). Huntington's disease (HD); Parkinson's disease (PD); healthy controls after splitting into groups matched to HD and PD: HC_{HD} and HC_{PD}.

Contrast	Brain region	Side	MNI (mm)			Local maximum t
			x	y	z	
$(HD - HC_{HD}) - (PD - HC_{PD})$	STN (left)	L	-10	-11	-7	3.11
	STN (right anterior)	R	8	-8	-10	3.97
	STN (right posterior)	R	12	-17	-7	3.35
$-(HD - HC_{HD}) + (PD - HC_{PD})$	Primary motor cortex (thigh)	L	-12	-30	68	5.59
	Primary somatosensory cortex (fist)	L	-39	-29	48	5.52
	Primary motor cortex (fist)	L	-42	-19	52	4.10
	Premotor cortex (fist/little finger)	L	-31	-13	61	4.06
	Primary somatosensory cortex	R	37	-44	61	4.39

Supplementary Table 4: Correlations between functional connectivity results and clinical and behavioural measures for Huntington's and Parkinson's disease groups. Correlation results between each supra-threshold resting-state functional MRI result in the sensorimotor cortex, substantia nigra (SN) and external globus pallidus (GPe) and clinical & cognitive measures, ordered according to increasing p-value. Scores are organised into motor (dark grey), cognitive (light grey) and other (white) items. Uncorrected p-values are reported, those surviving false discovery rate (FDR) correction at 10% are marked with ✓.

HD carriers

rsfMRI Group contrast	Brain region	UHDRS/HLVT item	Spearman's correlation Rho	p-value	FDR 0.1
$(HD - HC_{HD}) - (PD - HC_{PD})$	SN (left lateral)	Disease burden	0.64	4.04E-02	
		Bradykinesia-body	0.83	8.00E-04	✓
		Stroop colour naming errors	0.72	7.90E-03	
		Stroop word reading errors	0.59	4.29E-02	
	SN (left posterior)	Perseverative thinking/behaviour frequency	-0.81	1.30E-03	✓
		Perseverative thinking/behaviour severity	-0.61	3.54E-02	
	GPe (left)	Functional finances	-0.62	3.33E-02	
		Categorical verbal fluency test correct	-0.61	3.69E-02	
	GPe (right)	Saccade velocity, vertical	-0.77	3.50E-03	
		Hopkins verbal learning test delayed recall	0.64	2.55E-02	

PD patients

rsfMRI Group contrast	Brain region	UPDRS item	Spearman's correlation Rho	p-value	FDR 0.1
$(HD - HC_{HD}) - (PD - HC_{PD})$	SN (left posterior)	II - Chewing and swallowing	-0.66	3.82E-03	
		I - Apathy	-0.60	1.12E-02	
	GPe (left)	III - Sum motor scores, right *	-0.65	5.03E-03	
		III - Asymmetry groups	0.55	2.35E-02	
		II - Hobbies activities	-0.55	2.12E-02	
		IV - Functional impact of fluctuations	-0.51	3.45E-02	
		IV - Time spent in the "off" state	-0.50	3.91E-02	
		IV - Complexity of motor fluctuations	-0.48	4.97E-02	
		I - Fatigue	-0.56	1.89E-02	
	GPe (right)	II - Dressing	0.65	4.46E-03	
		I - Daytime sleepiness	-0.49	4.42E-02	

* "III - Sum motor score, right" is the sum of all the left lateralised motor scores in UPDRS-III.
When rs-fMRI results were not significant according to FDR correction, weighted averages extracted from uncorrected-p<0.001 clusters of interest were considered for correlations.
